# Targeted next-generation sequencing for genetic variants of left ventricular mass status among community-based adults in Taiwan

**DOI:** 10.3389/fgene.2022.1064980

**Published:** 2023-01-12

**Authors:** Hsien-Yu Fan, Wan-Yu Lin, Tzu-Pin Lu, Yun-Yu Chen, Justin BoKai Hsu, Sung-Liang Yu, Ta-Chen Su, Hung-Ju Lin, Yang-Ching Chen, Kuo-Liong Chien

**Affiliations:** ^1^ Institute of Epidemiology and Preventive Medicine, National Taiwan University, Taipei, Taiwan; ^2^ Department of Family Medicine, School of Medicine, College of Medicine, Taipei Medical University, Taipei, Taiwan; ^3^ Department of Medical Research, Taichung Veterans General Hospital, Taichung, Taiwan; ^4^ Cardiovascular Center, Taichung Veterans General Hospital, Taichung, Taiwan; ^5^ Heart Rhythm Center, Division of Cardiology, Department of Medicine, Taipei Veterans General Hospital, Taipei, Taiwan; ^6^ Cardiovascular Research Center, School of Medicine, National Yang Ming Chiao Tung University, Taipei, Taiwan; ^7^ Department of Computer Science and Engineering, Yuan Ze University, Taoyuan, Taiwan; ^8^ Department of Clinical Laboratory Sciences and Medical Biotechnology, College of Medicine, Taipei, Taiwan; ^9^ Department of Internal Medicine, National Taiwan University Hospital, Taipei, Taiwan; ^10^ Department of Family Medicine, Taipei Medical University Hospital, Taipei Medical University, Taipei, Taiwan; ^11^ School of Nutrition and Health Sciences, College of Nutrition, Taipei Medical University, Taipei, Taiwan; ^12^ Graduate Institute of Metabolism and Obesity Sciences, Taipei Medical University, Taipei, Taiwan

**Keywords:** next-generation sequencing, left ventricular mass, genetic variants, gene expression, gene network

## Abstract

**Background:** Left ventricular mass is a highly heritable disease. Previous studies have suggested common genetic variants to be associated with left ventricular mass; however, the roles of rare variants are still unknown. We performed targeted next-generation sequencing using the TruSight Cardio panel, which provides comprehensive coverage of 175 genes with known associations to 17 inherited cardiac conditions.

**Methods:** We conducted next-generation sequencing using the Illumina TruSight Cardiomyopathy Target Genes platform using the 5% and 95% extreme values of left ventricular mass from community-based participants. After removing poor-quality next-generation sequencing subjects, including call rate <98% and Mendelian errors, 144 participants were used for the analysis. We performed downstream analysis, including quality control, alignment, coverage length, and annotation; after setting filtering criteria for depths more than 60, we found a total of 144 samples and 165 target genes for further analysis.

**Results:** Of the 12,287 autosomal variants, most had minor allele frequencies of <1% (rare frequency), and variants had minor allele frequencies ranging from 1% to 5%. In the multi-allele variant analyses, 16 loci in 15 genes were significant using the false discovery rate of less than .1. In addition, gene-based analyses using continuous and binary outcomes showed that three genes (*CASQ2*, *COL5A1*, and *FXN*) remained to be associated with left ventricular mass status. One single-nucleotide polymorphism (rs7538337) was enriched for the *CASQ2* gene expressed in aorta artery (*p* = 4.6 × 10–18), as was another single-nucleotide polymorphism (rs11103536) for the *COL5A1* gene expressed in aorta artery (*p* = 2.0 × 10–9). Among the novel genes discovered, *CASQ2*, *COL5A1*, and *FXN* are within a protein–protein interaction network with known cardiovascular genes.

**Conclusion:** We clearly demonstrated candidate genes to be associated with left ventricular mass. Further studies to characterize the target genes and variants for their functional mechanisms are warranted.

## 1 Introduction

Cardiovascular diseases (CVDs), principally coronary artery diseases and stroke, are the most common causes of death worldwide and a major contributor to disability (Roth et al., 2020). Early diagnosis and treatment of CVDs increases the chances of survival (Akyea et al., 2020). For patients with CVDs, the priority is to prevent subsequent CVD events, including myocardial infarctio, stroke, and death. Secondary prevention strategies for patients with CVDs have achieved success in reducing the mortality and morbidity from the incident CVD (Chen et al., 2015).

Left ventricular mass is a well-established measure that can predict adverse prognosis in CVDs (Tsao et al., 2015). An increased mass of the left ventricle serves initially as a compensatory response by the heart to increases in cardiac afterload (Lorell and Carabello, 2000). The increased left ventricular mass frequently becomes injury or maladaptive over time, contributing to diastolic dysfunction, systolic dysfunction, symptomatic left-ventricular systolic and diastolic dysfunction, and death from CVD (Lorell and Carabello, 2000). Because the weight of the left ventricle is a highly heritable trait, genetic factors play important roles in left ventricular mass (Chien et al., 2006), for thier novel mutation, copy number variations, or differences in maternally inherited mitochondrial deoxyribonucleic acid (DNA) with varied even within an individual.

Previous studies have suggested that genetic variants associated with left ventricular mass are from common variants, and these variants have been genotyped using genome-wide association study arrays (Arnett et al., 2009; Sarwar and Cook, 2009; Bella and Goring, 2012; Barve et al., 2016; van der Harst et al., 2016; Aung et al., 2019). However, data from next-generation sequencing platforms, such as targeted sequencing, have been scanty, especially from community participants. Therefore, we conducted targeted sequencing to examine genetic variants for the echocardiographically determined left ventricular mass from a community-based cohort, using the extreme value design.

## 2 Manuscript formatting

### 2.1 Methods

#### 2.1.1 Study design and population

As shown in the study flow chart (Supplementary Figure S1), we collected two extreme groups of people with adjusted left ventricular mass. The adjusted variables included age, gender, body mass index, systolic blood pressure, smoking, drinking, and exercise status. The adjusted R-square was .28 and the residual left ventricular mass in each participant was added to the sample average value (193.2 gm). After completing the selection of extreme-pair samples and DNA preparations, we prepared a total of 144 participants’ DNA samples for further bioinformatics and biostatistics analyses.

A total of 276 Taiwanese adults underwent echocardiographic examination, approved by the Institutional Research Board of the National Taiwan University Hospital (NTUH-REC no. 201612177RINA). We confirmed that all experiments were undertaken in accordance with the relevant guidelines and regulations.

#### 2.1.2 Laboratory measurements

In total, 144 participants were genotyped using the TruSight Cardio Kit sequencing array developed by Illumina (San Diego, CA). After removing poor-quality NGS subjects, including call rate <98% and Mendelian errors, 144 participants were used for analysis. We performed the NGS genotyping platform using the TruSeq Exome Enrichment Kit (Illumina, San Diego, CA); the secondary analyses were performed using Illumina TruSight Cardiomyopathy Target Genes. We used the parameters species: *Homo sapiens*, genome builds: GRCh38.94, total samples: 144, library preparation: TruSightTM Cardio Sequencing Kit. We analyzed a total of 174 genes with known associations to 17 different inherited cardiac conditions, and we used the related software for the data analytic pipeline (Supplementary Table S1). We performed the downstream analyses and confirmed that the related quality control parameters were good (Supplementary Appendix Page 2; Supplementary Figures S2–S10; Supplementary Table S2). We applied the recommended criteria as the suggested Q30 value >75% and duplicated reads/total reads <30%. We resorted to multi-marker analyses. Because the SNP positions of the data were based on the human genome (GRCh38. p12, Genome Reference Consortium, hg38 database) assembly, we mapped variants into genes according to the same assembly (https://asia.ensembl.org/Homo_sapiens/Info/Index). The 5′ and 3′ flanking regions of each gene were included in this study. For example, a promoter is the 5′ flanking region of DNA where transcription of the gene is initiated. The region for transcription termination was in the 3′ flanking region of the gene. The related details of quality control and data process are listed in the Supplementary Method Section.

#### 2.1.3 Statistical analysis

We adjusted for age, gender, body mass index, and systolic blood pressure as the covariates in the binary trait (≥95th percentiles vs. ≤5th percentiles) and continuous variables (original and adjusted left ventricular mass values). We defined two extreme groups as participants beyond the 5th and 95th percentiles of adjusted left ventricular mass in one community. For example, cases (left ventricular hypertrophy) are defined as having a left ventricular mass index >95th percentile. Controls are defined as having a left ventricular mass index <5th percentile.

First, we treated the outcomes using binary status (extreme high and low groups) and continuous variables (original left ventricular mass and adjusted left ventricular mass). Second, we used the simple and multiple covariate-adjusted models. The adjusted covariates in the model included age, gender, body mass index, and systolic blood pressure. We used dummy variables for age, body mass index, and systolic blood pressure values in the model to handle the non-linearity relationship of the covariates. We defined the following confounding variables as gender (men or women), smoking (yes/no or abstinence), drinking (regular/no), regular physical activity (yes/no), age (years), blood pressure (mmHg), body mass index (kg/m^2^).

#### 2.1.4 Variant and gene-based analyses

First, we performed the single-variant-based analysis, testing the additive, dominant, and recessive modes of inheritance. Treating the left ventricular mass as the continuous variable and the binary variable, we performed general linear regression and logistic regression models to estimate the effect sizes of the variants for the outcomes. To adjust for multiple comparison, we used the Bonferroni correction and the false discovery rate (FDR) by the BH (Benjamini–Hochberg) procedure to control Type I error (Benjamini and Hochberg, 1995). Next, we performed gene-based analysis using the sequence kernel association test–optimal (SKATO) method (Wu et al., 2011). To reduce noise from the association signals of multiple markers within a gene, we also performed an adaptive combination of Bayes factors (ADABF) method (Lin et al., 2014; Lin et al., 2018). When we considered both rare and common variants, the ADABF method was more robust in the inclusion of neutral variants than was the SKATO method (Lin et al., 2017). The ADABF approach applied the sequential resampling strategy, in which the minimum and maximum numbers of resampling were set as 10^4^ and 10^8^, respectively (Lin et al., 2014), and the results of SKATO and ADABF methods may show that both dense and sparse causal variants could appear in different regions across the genome. We also plotted the Manhattan plot of overall genome level. We used the program R (version 3.5.3) to perform the genetic statistical analysis.

#### 2.1.5 Gene expression

Expression quantitative trait loci (eQTL) analysis was performed using the Genotype–Tissue Expression (GTEx) consortium (Lonsdale et al., 2013); version 8 (v8) includes genotype data from 948 donors and RNA-seq samples across 54 tissue sites. Briefly, genotyping was performed using HiSeq 2000 (e.g., HiSeq X, Agilent, or ICE target capture) and Illumina Array (e.g., OMNI 5M Array or 2.5M SNP, Human Exome SNP). The Illumina TruSeq library construction protocol was used to perform ribonucleic acid (RNA) sequencing. The normalized effect size for each SNP from the eQTL summary data is defined as the slope of the linear regression. The normalized effect size of the eQTL is computed from the effect of the alternative (ALT) allele relative to the reference allele (REF) in the genome reference, based on quantile standardized expression data (Mohammadi et al., 2017). For example, a multi-tissue eQTL plot for rs6732341 was calculated in the GTEx browser. It was computed as the effect of the alternative allele C relative to the reference allele T in rs10157905 (chr2_629817_C_T_b38) of *CASQ2* gene in the reference human genome GRCh38/hg38. The analysis was limited to tissues determined *a priori* to be relevant to cardiovascular-related traits. For comparison, the *p*-values for each identified tissue were listed for all SNPs, as well as the minimum *p*-values across all SNPs.

#### 2.1.6 Genetic correlations among implicated loci

We used the inBio Map™ database of high-confidence protein–protein interactions to explore the genetic relatedness among implicated genes (Li et al., 2017). The input consisted of three genes (*CASQ2*, *COL5A1*, and *FXN*) as identified in the analyses and generated the network with a confidence cutoff of .02.

### 2.2 Results

#### 2.2.1 Demographic characteristics

The study flow chart is described in Supplementary Figure S1, shown in the online supplement. The study participants underwent echocardiographic examinations, and we estimated the left ventricular mass of all participants. Using extreme distribution (i.e., <5th and >95th percentile values), we collected 144 subjects with full information and all genetic information available. We compared the basic characteristics between these two groups (*n* = 276) and found that compared with those in the low extreme group (<5th percentile), the participants in the high extreme group (>95th percentile) were likely to be men and to have smoking and drinking histories (Table 1). However, the distributions of age, systolic blood pressure, and body mass index were similar between two groups.

**TABLE 1:** Basic characteristics of the study participants, stratified by the status of extreme groups of adjusted center ventricular mass (<5% vs. >95%).

Characteristic		>5% percentile		>95% percentile		*p*-value
		N (%)	Mean (SD)	N (%)	Mean (SD)	
Gender	Men	88 (63.8)		68 (49.3)		.02
	Women	50 (36.2)		70 (50.7)		
Smoking	No	69 (50)		85 (61.6)		.07
	Yes	69 (50)		53 (38.4)		
Drinking	No	83 (60.1)		101 (73.2)		.03
	Yes	55 (39.9)		37 (26.8)		
Regular physical activity	No	109 (79)		117 (84.8)		.27
	Yes	29 (21)		21 (15.2)		
Age	Years		57.7 (12.4)		57.9 (10.6)	.90
Systolic blood pressure	mmHg		133 (20.9)		131.4 (23.5)	.55
Body mass index	kg/m^2^		24.8 (4.2)		24.4 (3.5)	.40
Left ventricular mass	gm		121.8 (28.1)		365.8 (74.8)	<.001
Adjusted center ventricular mass^a^	gm		94.8 (16.4)		349.1 (54.5)	<.001

^a^ Adjusted variables included age, gender, body mass index, systolic blood pressure, smoking, drinking, and exercise status. The adjusted R-square was .28, and the residual center ventricular mass in each participant was added to the sample average value (193.2 gm).

#### 2.2.2 Identification of target genes in left ventricular mass status

We showed a typical L-shaped distribution of minor allele frequencies (MAFs) of variants in the study participants (left figure in Supplementary Figure S11) and limited in MAF <.10 (right figure). The Manhattan plot of all loci, by the four methods at the genome-wide level in all study participants, is shown in Supplementary Figure S12.

Based on an FDR less than .05, we identified 96 loci for left ventricular mass. To estimate the number of independent loci, we pruned the associated SNPs with LD > .2 and identified a total of 16 independent SNPs in *CASQ2*, *APOB*, *LAMA4*, *DSP*, *COL5A1*, *FXN*, *RBM20*, *ILK*, *ABCC9*, *CACNA1C*, *FBN1*, *TPM1*, *RYR1*, *RYR1*, *TXNRD2*, and *SCO2* (Table 2). Next, we conducted the gene-based analyses using the continuous and binary outcomes of both the SKATO and the ADABF methods in additive modes of inheritance (Table 3). We found that under the SKATO method, nine genes were marginally associated with left ventricular mass status, and under the ADABF method, six genes were associated with left ventricular mass status. We highlighted three overlapping genes (*CASQ2*, *COL5A1*, and *FXN*) based on both single-variant and gene-based analyses.

**TABLE 2:** Minimal *p*- and *q*-values in the single-variant analysis for the study participants.

Gene name	rsID	Chromosome: position	Reference allele	Effect allele	Minor allele frequency	Estimate	Standard error	*p*-value	*q* value*
*CASQ2*	rs7538337	1:115740905	G	A	.186	74.08	20.46	3.5E-04	.09
*APOB*	rs140027955	2:21009645	G	A	.003	467.81	133.21	9.1E-04	.09
*LAMA4*	rs78253477	6:112141551	T	C	.007	342.42	94.26	5.0E-04	.09
*DSP*	rs139799237	6:7576437	G	A	.003	467.81	133.21	9.1E-04	.09
*COL5A1*	rs11103536	9:134809545	G	A	.476	58.17	16.23	4.6E-04	.09
*FXN*	rs563239532	9:69036038	C	G	.003	467.81	133.21	9.1E-04	.09
*RBM20*	rs142673498	10:110727905	G	A	.007	233.90	66.61	9.1E-04	.09
*ILK*	rs199789449	11:6604307	C	T	.003	467.81	133.21	9.1E-04	.09
*ABCC9*	rs1492135	12:21907922	T	C	.007	233.90	66.61	9.1E-04	.09
*CACNA1C*	rs566615019	12:2633412	C	T	.003	467.81	133.21	9.1E-04	.09
*FBN1*	rs1289016843	15:48503507	T	C	.003	467.81	133.21	9.1E-04	.09
*TPM1*	rs28583444	15:63065653	T	G	.007	233.90	66.61	9.1E-04	.09
*RYR1*	rs4802515	19:38503436	T	C	.021	154.37	42.38	9.5E-04	.09
*RYR1*	rs200813231	19:38543675	C	T	.003	467.81	133.21	9.1E-04	.09
*TXNRD2*	rs143966577	22:19883313	C	T	.003	467.81	133.21	9.1E-04	.09
*SCO2*	rs201909075	22:50523836	G	A	.003	467.81	133.21	9.1E-04	.09

*The false discovery rate (FDR) was used to control Type I error.

Models were adjusted for age, sex, body mass index, and systolic blood pressure.

**TABLE 3:** Summarized significance level (*q*-value of .05) for gene-based results according to the additive modes of inheritance.

Gene name	CHR^a^	Position	SKATO		ADABF	
			Binary outcome	Continuous outcome	Binary outcome	Continuous outcome
*FXN*	9	69035752-69079076	.01	.08	.05	.03
*GCKR*	2	27496839-27523684	.02	.01	.04	.13
*APOE*	19	44905796-44909393	.03	.03	.05	.15
*NODAL*	10	70431936-70441681	.03	.04	.11	.11
*KRAS*	12	25205246-25250929	.04	.01	.39	.19
*LMF1*	16	853634-970984	.00	.01	.36	.20
*RBM20*	10	110644336-110839468	.01	.00	.35	.21
*MYPN*	10	68106123-68211703	.05	.03	.43	.30
*ZHX3*	20	41178455-41317731	.01	.01	.93	.66
*COL5A1*	9	134641803-134844843	.32	.72	.02	.01
*GJA5*	1	147756200-147773362	.05	.06	.02	.01
*FKRP*	19	46746057-46758575	.73	.75	.01	.02
*GATAD1*	7	92447482-92460075	.60	.55	.01	.03
*CASQ2*	1	115700021-115768714	.47	.87	.03	.03
*FHL2*	2	105360826-105438503	.99	.90	.05	.04

^a^ CHR, chromosome; SKATO, sequence kernel association test–optimal method; ADABF, adaptive combination of Bayes factors method.

The summarized effect sizes for adjusted left ventricular mass and extreme status under various modes of inheritance, and the frequency of selected variants and genes between the case and control groups in the study participants, are listed in Supplementary Tables S3, S4. When considering the status of extreme case/control status and under the additive mode, the effect size of the variant rs11103537, in the *COL5A1* gene, was −3.6 (*p* = .0003), and the variant frequency for control was 39.4% and for case was 16.9%. When considering the adjusted left ventricular mass, the effect size of the variant rs142673498, in the *RBM20* gene, was 3.4 (*p* = .001), with the frequency for the control group of less than .001 and for the case of 1.5%.

#### 2.2.3 Expression quantitative trait loci effects related to left ventricular mass

We list expression of quantitative trait loci effects related to left ventricular mass based on the minimum *p*-value (Table 4). One single-nucleotide polymorphism (rs7538337) was enriched for the *CASQ2* gene expressed in the aorta artery (*p* = 4.6 × 10^–18^); another single-nucleotide polymorphism (rs11103536) was enriched for the *COL5A1* gene expressed in the aorta artery (*p* = 2.0 × 10^–9^).

**TABLE 4:** Overview of expression quantitative trait loci (eQTL) effects related to center ventricular mass.

Gene name	rsID	MAF	Risk allele/reference allele	Chromosome: position	Tissue	Effect size	Minimum *p*-value
*CASQ2*	rs10157905	.50	C/T	1:115765890	Aorta artery	.28	2.7E-49
*COL5A1*	rs13294483	.33	G/A	9:134784148	Never artery	.21	1.9E-9
*FXN*	rs7039631	.43	G/A	9:69078376	Skeletal muscle	−.17	2.5E-16

eQTL, expression assay based on the GTEx portal expression data; FDR, false discovery rate; MAF, minor allele frequency; N.A., not applicable.

#### 2.2.4 Functional gene network for the targeted genes

**FIGURE 1:**
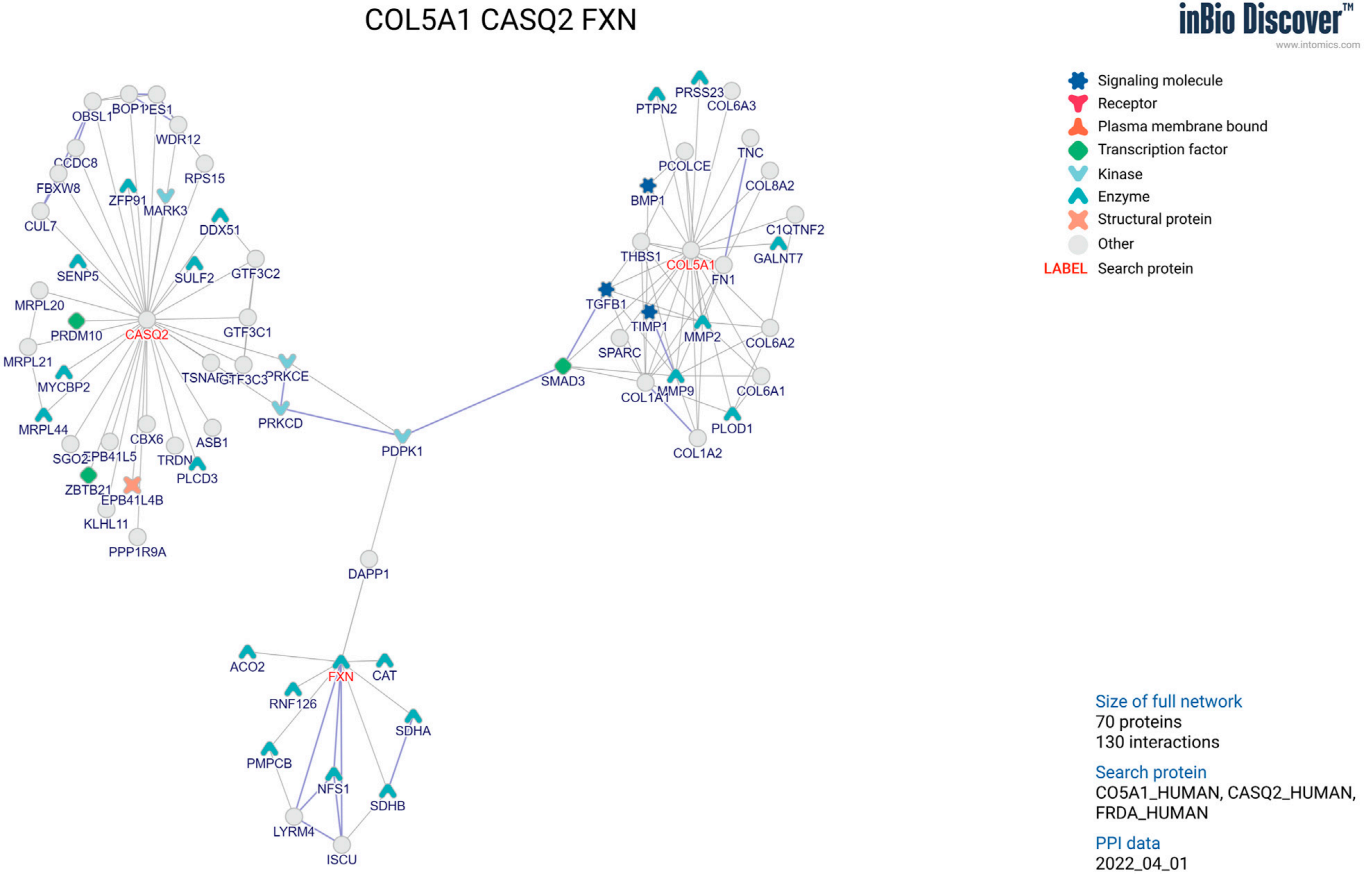
Functional gene network for the targeted genes from the present analysis.

We show the functional gene network for the targeted loci in Figure 1. Among the novel genes discovered, *CASQ2*, *COL5A1*, and *FXN* are within the protein–protein interaction network of known cardiovascular genes.

### 2.3 Discussion

#### 2.3.1 Main findings

We clearly demonstrated cardiomyopathy candidate genes to be associated with left ventricular mass. Among the novel genes discovered, *CASQ2*, *COL5A1*, and *FXN* are within the protein–protein interaction network of known cardiovascular genes.

#### 2.3.2 Comparison of the current study with previous studies

Compared with GWAS study findings for matches and differences (Aung et al., 2019), we found only two target genes (*TTN* and *BAG3*) within the TruSight Cardio list. However, our gene-based analyses showed that these two genes may not be associated with left ventricular mass status, possibly due to differences in race or ethnicity. In addition, other previous studies have provided evidence from the candidate genes and genome-wide association approaches (Arnett et al., 2009; Sarwar and Cook, 2009; Bella and Goring, 2012; Barve et al., 2016; van der Harst et al., 2016). However, the current literature was restricted to hospital-based data and did not focus on community-based adults; in addition, the modes of inheritance, including additive, dominant, and recessive modes, were not extensively tested. We vigorously investigated various modes of inheritance and used novel gene-based approaches, including the SKATO and ADABF methods. Our data clearly demonstrate that several variants, especially in specific genes, play important roles in left ventricular mass.

#### 2.3.3 The proposed biological mechanism

Our study provides suggestive evidence for the involvement of these genes in the trait studied. For example, expression levels in the aorta artery might be the mechanism underlying the *CASQ2* gene and left ventricular mass. Notably, the *CASQ2* gene has been reported to be a high-capacity Ca-binding protein expressed inside the sarcoplasmic reticulum (Györke et al., 2004). The *CASQ2* gene is an overlap gene which plays a key role in cardiomyocyte calcium handling related to muscle contraction (Tadros et al., 2021). *COL5A1* expression in nerve artery may affect left ventricular mass. This finding can be verified by a previous study indicating that *COL5A1* expression significantly correlates with the cardiac traits of chamber size, left ventricular mass, and systolic and diastolic function (Yokota et al., 2020). In addition, the *FXN* gene might be associated with left ventricular mass. This finding seems in line with a previous study describing the *FXN* gene, which was associated with structural and functional left ventricular changes (Peverill et al., 2019). For example, an increased *FXN* severity was associated with a smaller left ventricle and increased left ventricle wall thickness in adults but was not associated with left ventricle size or wall thickness in children (Peverill et al., 2019). However, the *CASQ2*, *COL5A1*, and *FXN* genes have been reported separately and have not been linked together. Therefore, we utilized the inBio website to explore the possible interaction network among the three key genes. As shown in Figure 1, we added a molecular pathway underlying the *PDPK1* gene as a central kinase linking the three genes. The results imply that the genes may have important functional impacts in cardiac muscle and development.

#### 2.3.4 Clinical and public health implications

Based on the finding, we propose genetic profiling for further screening of left ventricular mass status. First, we recommend that the identified genes and variants be used to construct polygenic risk scores to identify high-risk groups. Second, we provide precision medicine evidence-based data to explore the disease pathogenesis of left ventricular hypertrophy and related cardiomyopathy status. We reported three candidate genes that not only enhance our understanding of the genetic architecture of prognostically important left ventricular phenotypes but also shed light on a potential molecular screening tool for left ventricular systolic dysfunction.

#### 2.3.5 Study strength and weakness

To the best of our knowledge, this is the first study to apply the next-generation sequencing platform to investigate left ventricular mass. Using an extensive targeted-variant platform, we explored both common and rare variants at the same time. Second, we collected data from community-based adults, and the results have a broad community-based implication, with the echocardiographic measurements of left ventricular mass as a reliable indicator. Third, we adjusted multiple covariates, including age, gender, obesity, and blood pressure, and the residual effects are more attributed from the genetic factors. In addition, the strength of our study is that its use of two complementary approaches, each with different types of biases, improved the reliability of our findings. For example, from our results of the two complementary approaches, both dense and sparse causal variants appeared in different regions across the genome. Finally, the extra data (e.g., gene expression and gene network) generated from our study may facilitate novel strategies in precision medicine by increasing knowledge of the biological mechanisms influenced by targeted genes.

However, this study has several potential limitations. First, we did not explore the mediating effects of blood pressure and related drug usage on the association between the genetic variants and left ventricular mass. Second, we did not provide external validation data to test the applicability of our findings to other populations. Third, we remained focused on the targeted gene approach and did not investigate complete genomic information across the genome. Fourth, we also provided the Q-Q plot (Supplementary Figure S13) for the FDR or −log10(P) of these variants; as a result, the lambda statistic is not close to 1. There may exist false positives due to small sample size. Further approaches, such as whole-exome analysis, may provide additional information (Zhi et al., 2012). Finally, we did not perform functional assays to demonstrate the impacts of these variants on the related traits. As the TruSight Cardio panel targets 174 genes, we did not investigate other genes from other GWAS studies (e.g., UK Biobank) in our study. We also suggest that future studies are warranted to investigate whether other genes are associated with LV mass.

#### 2.3.6 Conclusion

In conclusion, our study provides substantial information about the roles of genetic variants on the determinants of left ventricular mass status and values. Further studies focusing on the biological mechanisms of specific genetic variants are warranted.

## Data Availability

The raw data supporting the conclusions of this article will be made available by the authors, without undue reservation. The original contributions presented in the study are included in the article/Supplementary Material; further inquiries can be directed to the corresponding author.
