# Supplementary material for: Targeted next-generation sequencing for genetic variants of left ventricular mass status among community-based adults in Taiwan
Source: Front Genet. 2023 Jan 12;13:1064980. doi: 10.3389/fgene.2022.1064980 (PMC9879005; doi:10.3389/fgene.2022.1064980)
Supplement: Supplementary file 1 [file DataSheet1.pdf]

**Online Supplement**

# Targeted next generation sequencing for genetic variants of left ventricular mass status among the community-based adults in Taiwan

**Hsien-Yu Fan, Wan-Yu Lin, Tzu-Pin Lu, Yun-Yu Chen, Justin BoKai Hsu, Sung-Liang Yu, Ta-Chen Su, Hung-Ju Lin, Yang-Ching Chen, Kuo-Liong Chien**

**Supplementary Materials ........ 3**
**Figure S1. The study flow chart of the study ........ 5**
**Figure S2. The depths for all samples are more than 30 ........ 6**
**Figure S3. FAST quality control for adapter content plot ........ 7**
**Figure S4. FAST Quality control for mean quality scores in the study samples ... 8**
**Figure S5. Per Sequence GC contents in the study samples ........ 9**
**Figure S6. The mean sequence quality by Phred scores in the study samples .....10**
**Figure S7. The proportions of Q30 values in the study samples ........11**
**Figure S8. The distribution of total sequences in the study samples ........12**
**Figure S9. Mapping rates of the study samples ........13**
**Figure S10. The average reads and the depths of the target genes and variants .14**
**Figure S11. The typical L-shaped distribution of minor allele frequencies (MAFs) of variants in the study participants (Left figure) and limited in MAF <0.10 (Right figure) ........15**
**Figure S12. Manhattan plot of the all SNPs by 4 methods in the all genome-wide level in the study participants ........16**
**Figure S13. The Q-Q plot for the FDR or -log10(P) of variants ........17**
**Figure S14. The summary plot histograms of the variant classification, variant types, SNV class, and the distribution of variants per sample, and the top 10 mutated genes in the study samples ........18**
**Figure S15. The onco-plot, onco-strip figures of the various functional mutations of the target genes in the study samples ........19**
**Figure S16. The plots for titv.png and TTN lolliplot and MYLK lolliplot (TiTv plot) ........20**
**Figure S17. The plots for titv.png and TTN lolliplot and MYLK lolliplot (the lollipop plot for one target gene, TTN) ........21**
**Figure S18. The plots for titv.png and TTN lolliplot and MYLK lolliplot (lollipop plot for one target gene, MYLK) ........22**

**Figure S19. The genecloud showed the proportions of the variants of target genes in the study samples........................................................................................23**
**Figure S20. The information about VCF file.....................................................24**
**Figure S21. The distribution of the variant frequency in each target gene in the study population (total 12287 variants and 175 genes) .......................................25**
**Table S1. The target genes and related frequency and percentage of the study participants..........................................................................................................26**
**Table S2. The summary table for the quality control of the study sample, by the average values of various indicators ...................................................................27**
**Table S3. Summarized effect size for the adjusted left ventricular mass, the frequency of selected variants and genes between the case and control groups in the study participants (Treating case/control group as the outcome)..................28**
**Table S4. Summarized effect size for the adjusted left ventricular mass, the frequency of selected variants and genes between the case and control groups in the study participants (Treating outcome as the adjusted left ventricular mass) 29**
**Table S5. The distribution of the functional change of variants in the study participants...........................................................................................................31**

## Supplementary Materials

### Downstream analyses and related quality control procedures

Re-call variants per sample using proper information of targeted genes, and merge individual calling result into one big matrix (n samples X m variants; two sub-steps in GATK4), then filter variants based on following criteria: keep variants with DP >=60, and replace missing data to reference, and split vcf into two files (SNP or Indel), then perform annotation and SNP Conversion, annotate variants and convert to snpMatrix (ATCG type) for further biostatistical analyses.

The following bioinformatics procedures for the quality assessment of the study samples:

Quality control: Our data quality is good, shown by the depths of sequencing are more than 30 **(Figure S2).** The FASTQC file adapter content plot showed a good pattern (**Figure S3**). And the mean quality score for FASTQC is good (**Figure S4**). We also checked the GC contents (%) in per sequence in our samples, and the results are acceptable (**Figure S5**). We checked the depths of the study samples, and found the mean sequence quality by Phred scores were good (**Figure S6)**.

The summary table for the quality control of the study sample, by the average values of various indicators, is the following **(Table S2)**: The average of Q30 was 86.44, and the average of duplicated Reads was 62.31%, with average GC content was 46.86%, indicating a good quality control. The proportions of Q30 values in the study samples were shown in **Figure S7**. In addition, **Figure S8** list the total sequences of the study samples, from $5.0x10^{6}$ to $2.0x10^{7}$, and the data provided sufficient sequences.

With regards to the alignment rates, our study samples showed an excellent alignment rates (>90%) (**Figure S9**). In addition, we checked the coverage rates using the average reads and the depths of the target genes and variants (**Figure S10**), and we found that the average reads depth were sufficient for further analysis

In selecting the variants, we set up the filtering criteria ad depth (DP)>=60, and our data showed that total raw detected variants among 145 samples: 2,514,185, total

remained detected variants among 145 samples: 55,294, total remained detected SNPs among 145 samples: 12,842. No remained detected SNPs within the 4 genes (*HRAS, KCNA5, ACTA1 and ACTC1*) under this filtering criteria (DP = 60) were found. We used the annotation databases including: refGene, avsnp150, ljb26_all (includes PolyPhen2 & SIFT), cosmic70, and exac03.

**Variant identification: and SNP Matrix file**

We checked the VCF file about the distributions of the SNPs, MNPs, Insertions, Deletions, Indels, and the missing Genotype, SNP transitions/transversions and total heterozygous/homozygous ratio in the study samples, and the data showed acceptable. Therefore, a total of 145 samples with 12842 variants are annotated successfully and we provided one SNP Matrix file for the variants' information, and the **Figure S14** showed the summary plot histograms of the variant classification, variant types, SNV class, and the distribution of variants per sample, and the top 10 mutated genes in the study samples, and the results were acceptable.

Next, we performed the annotation procedures, and the annotation of the variants in the study samples was successful in each target gene. **Figure S15** showed oncoplot, the onco-strip figures of the various functional mutations of the target genes in the study samples. For example, we checked the plots of for titv.png and *TTN* lolliplot were shown in **Figure S16** and the results were good. We provided the genecloud plot to see the proportions of the target gene variants in the study samples (**Figure S17**).

In addition, we provided the information about VCF file, using VCF representation for different types of variants: reference: 0/0, missing value: ./0, ./1,./., heterozygous: 0/1,1/2,0/2, homozygous: 1/1,2/2,3/3 in **Figure S18.**

After bioinformatics platform, we genotyped the 175 genes and a total of 12,287 variants, and the target genes and related frequency and percentage of the study participants in **Table S1**. The frequency distribution of variants was ranged from 0.01% in *CBS* gene to 5.8& in *TTN* gene**. Figure S19** showed that distributions of the variant frequency in each target gene in the study population. Multi-marker analysis showed the numbers of 609 of 3' UTR and 5'UTR, and 1804 exonic, 9875 intronic variants as the **Table S5.**

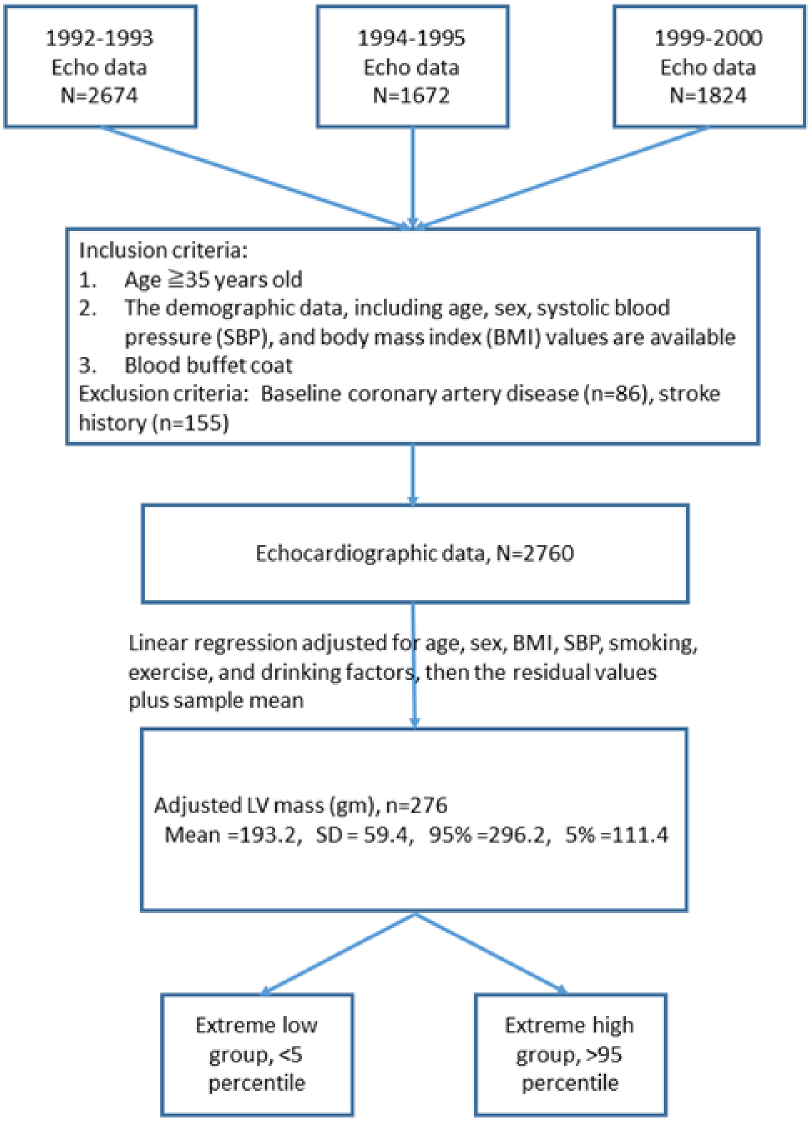


**Figure S1. The study flow chart of the study**

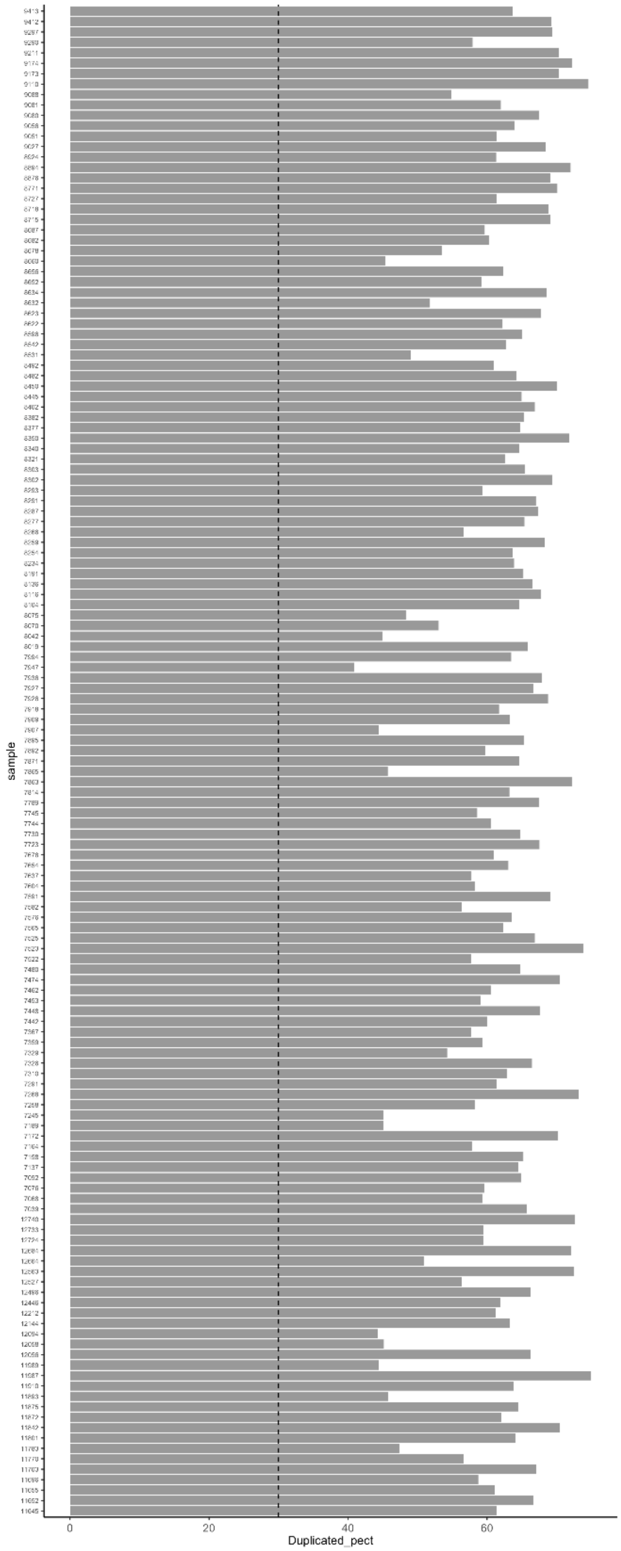


**Figure S2**. **The depths for all samples are more than 30**

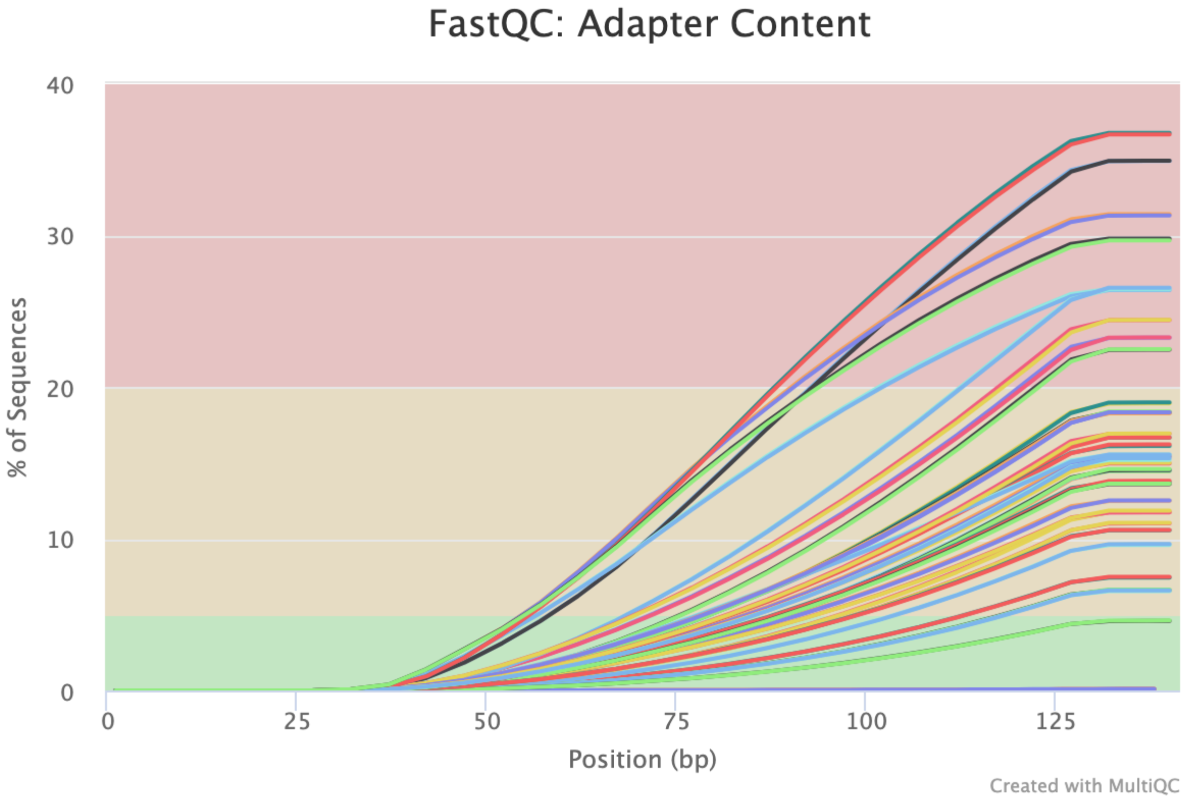


**Figure S3. FAST quality control for adapter content plot**

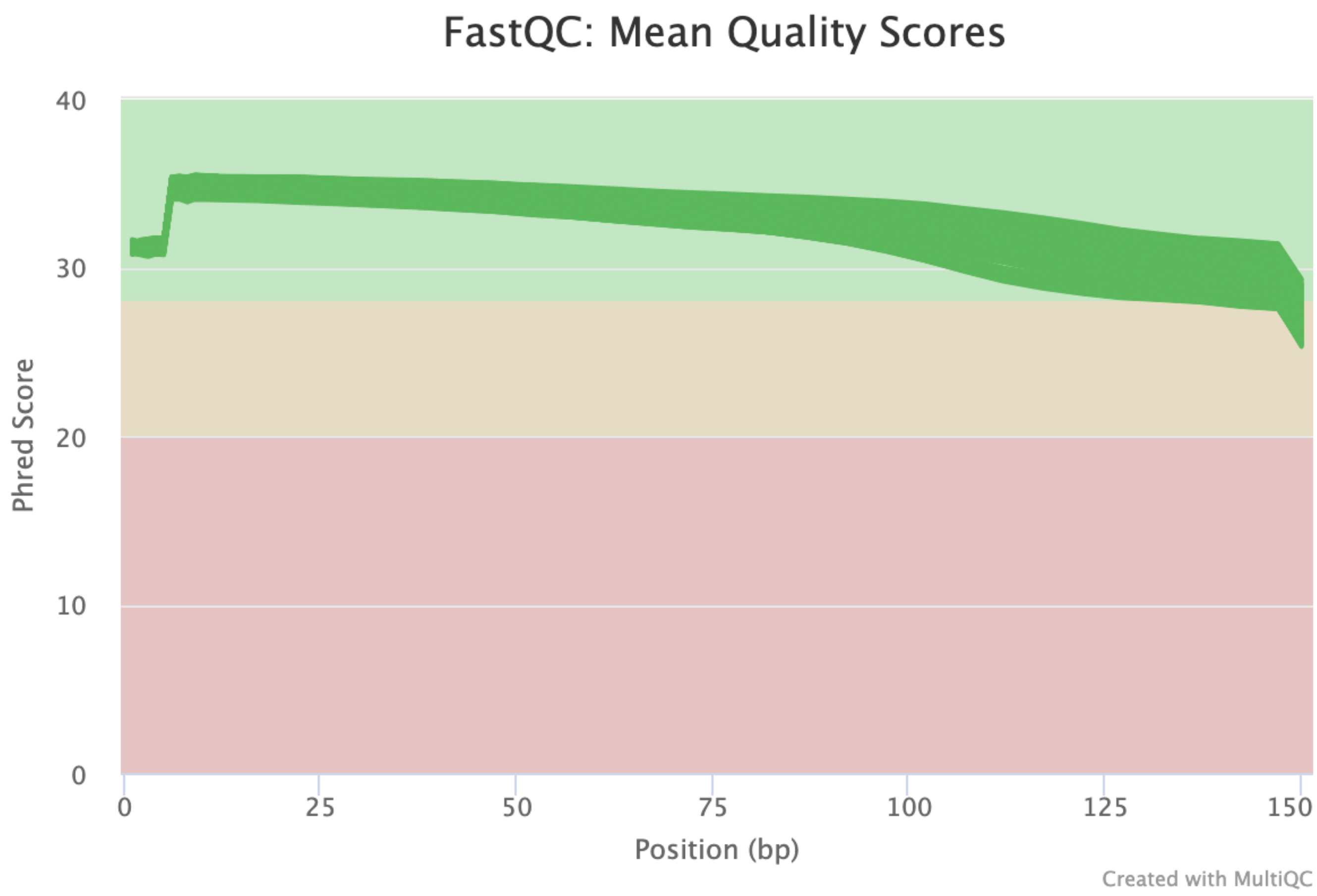


**Figure S4. FAST Quality control for mean quality scores in the study samples**

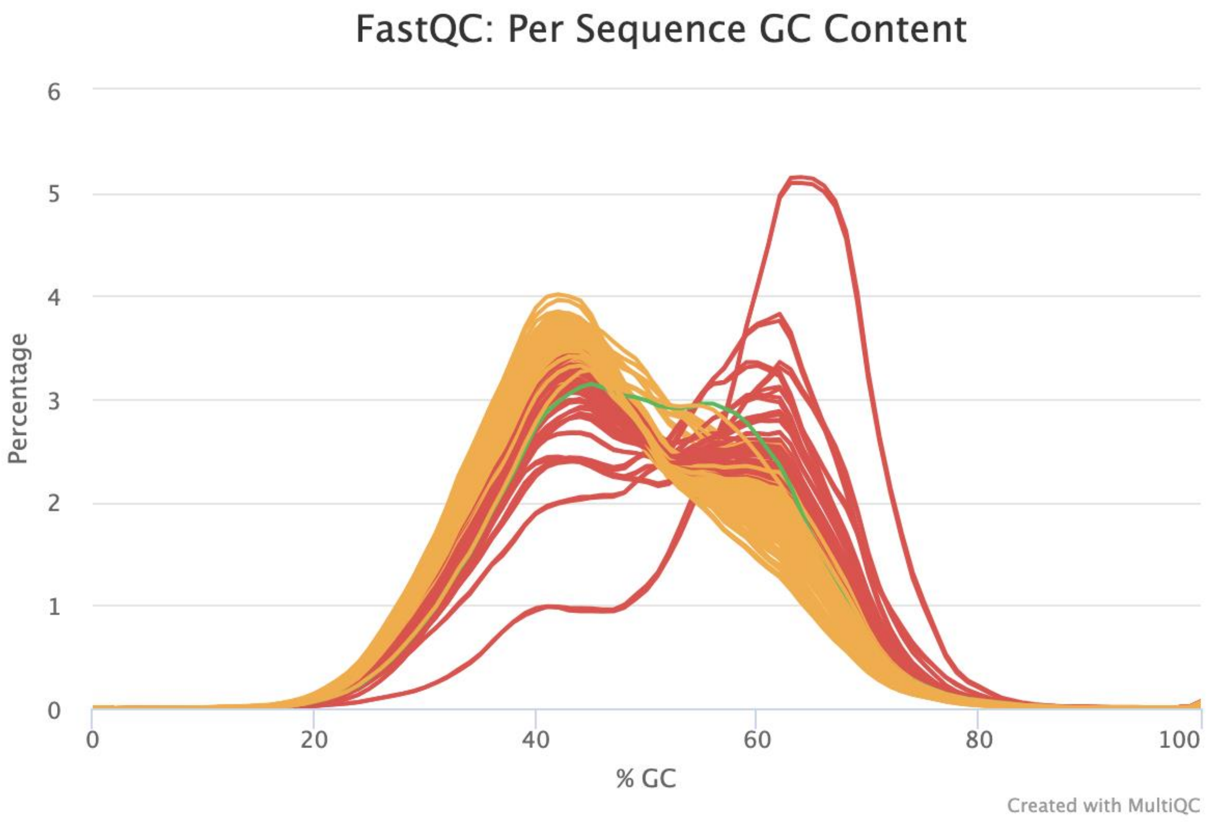


**Figure S5. Per Sequence GC contents in the study samples**

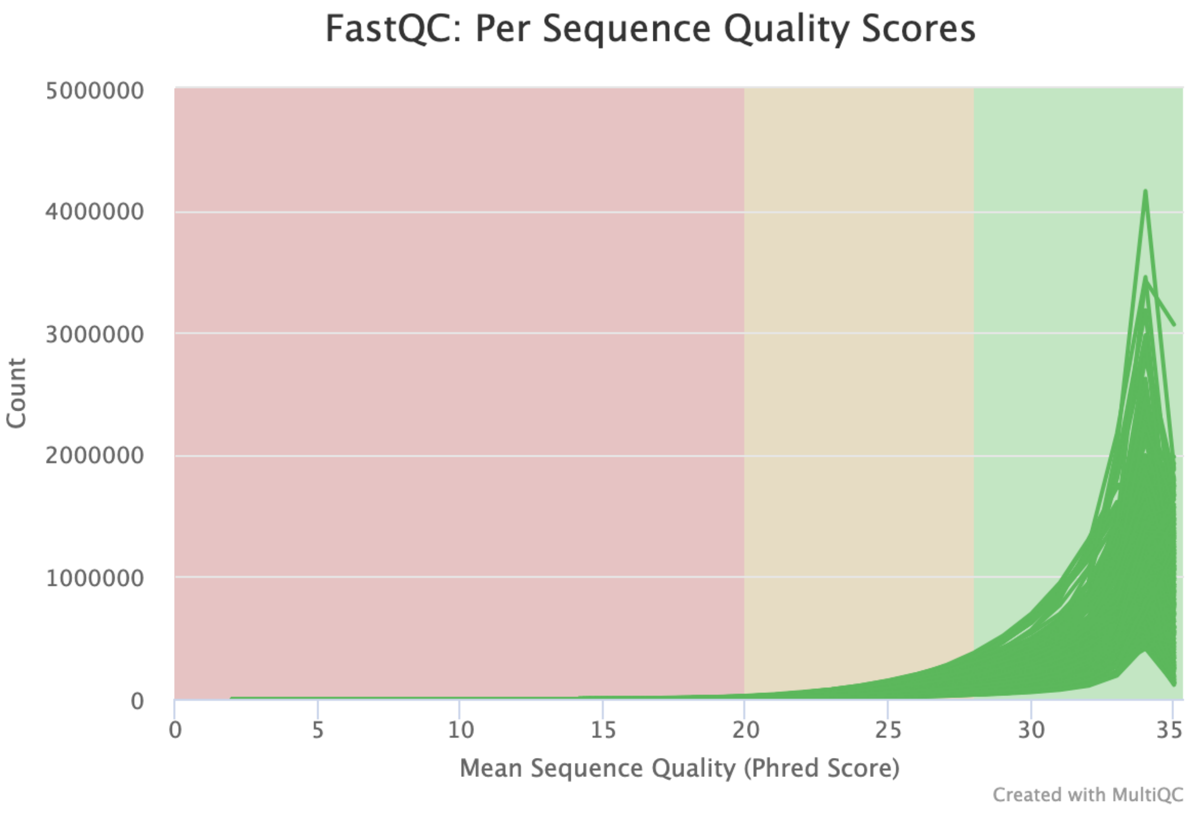


**Figure S6. The mean sequence quality by Phred scores in the study samples**

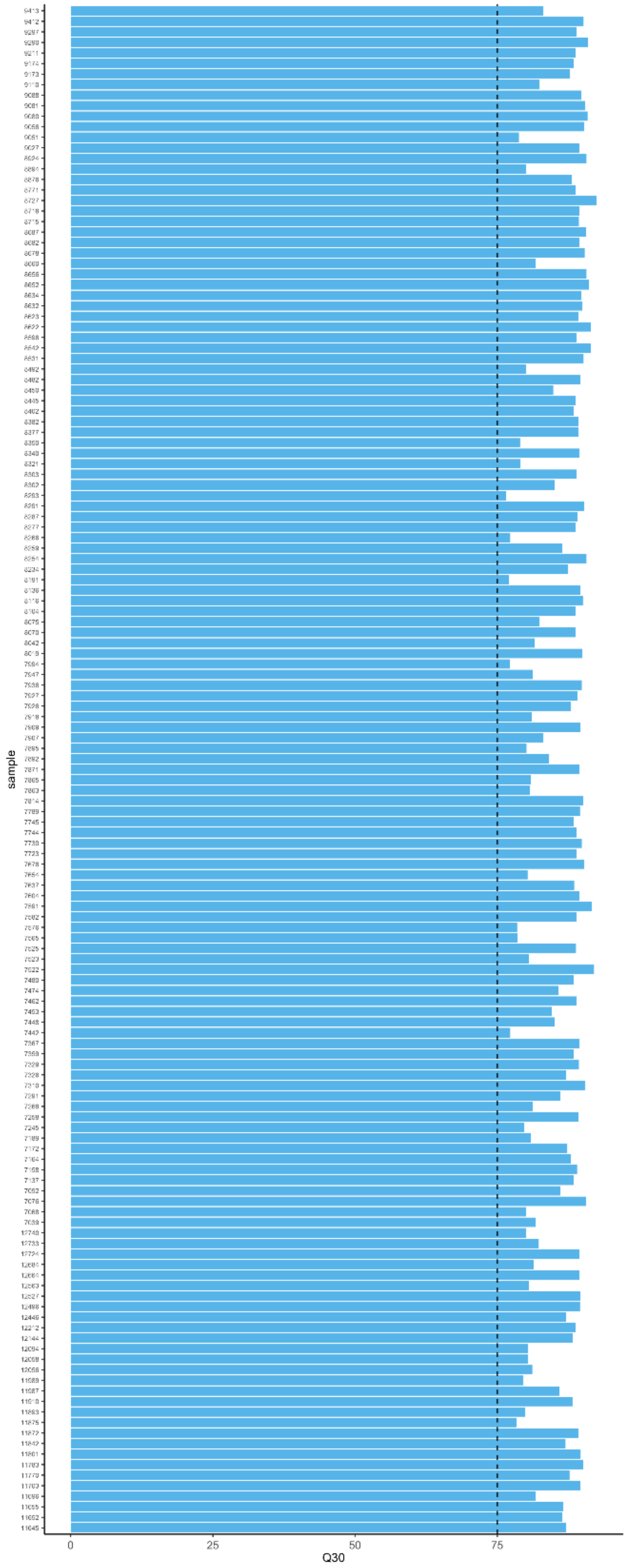


**Figure S7. The proportions of Q30 values in the study samples**

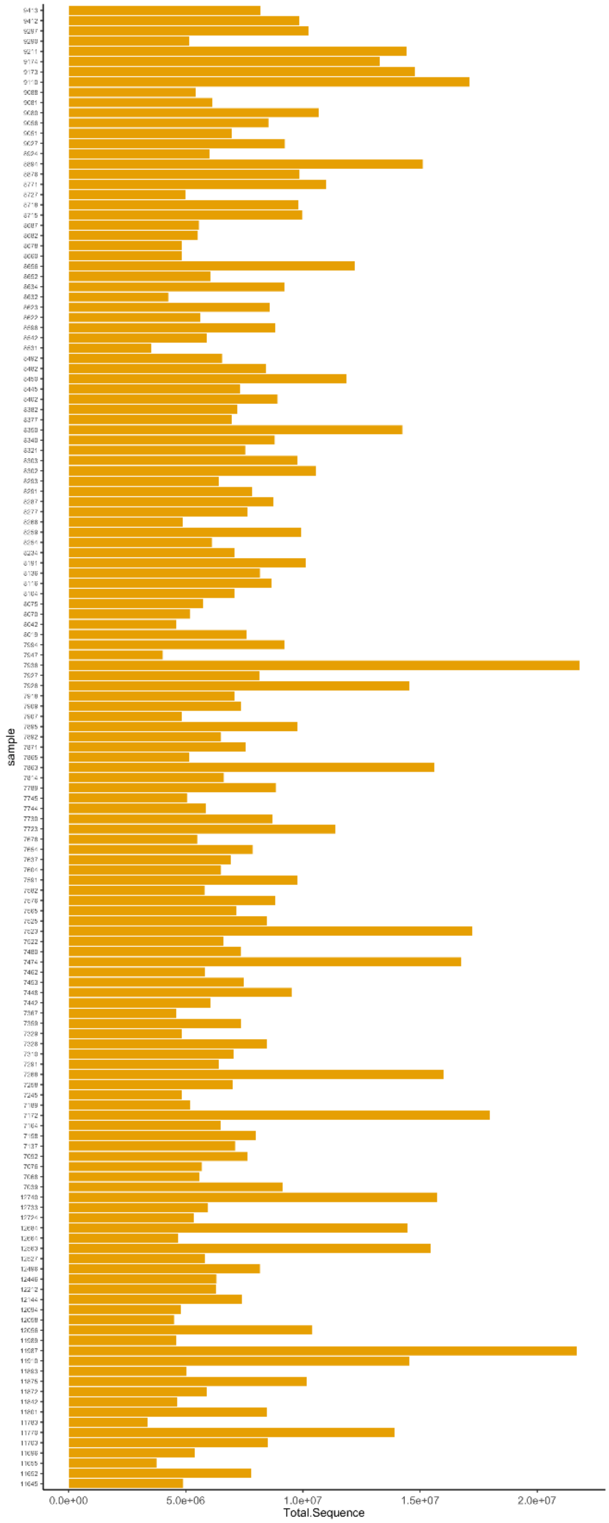


**Figure S8. The distribution of total sequences in the study samples**

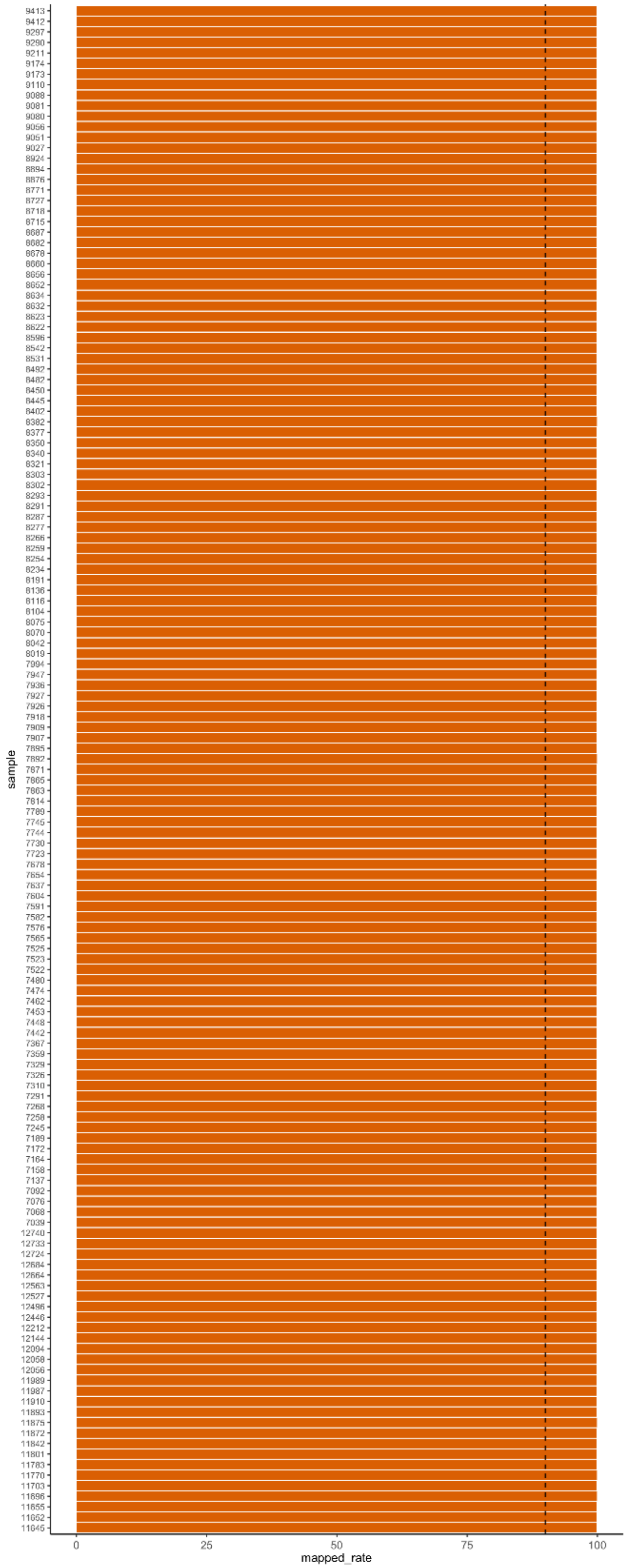


**Figure S9. Mapping rates of the study samples**

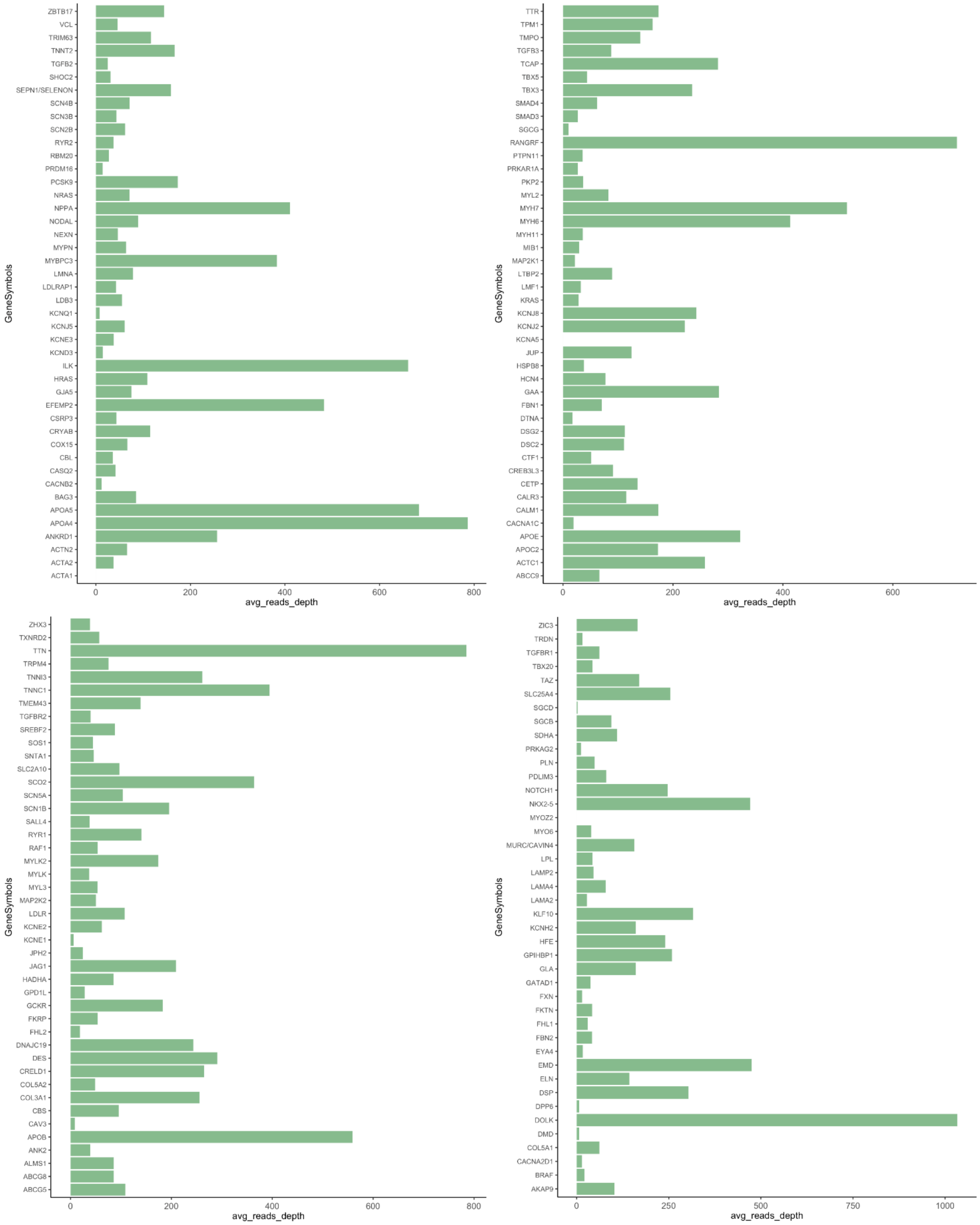


**Figure S10. The average reads and the depths of the target genes and variants**

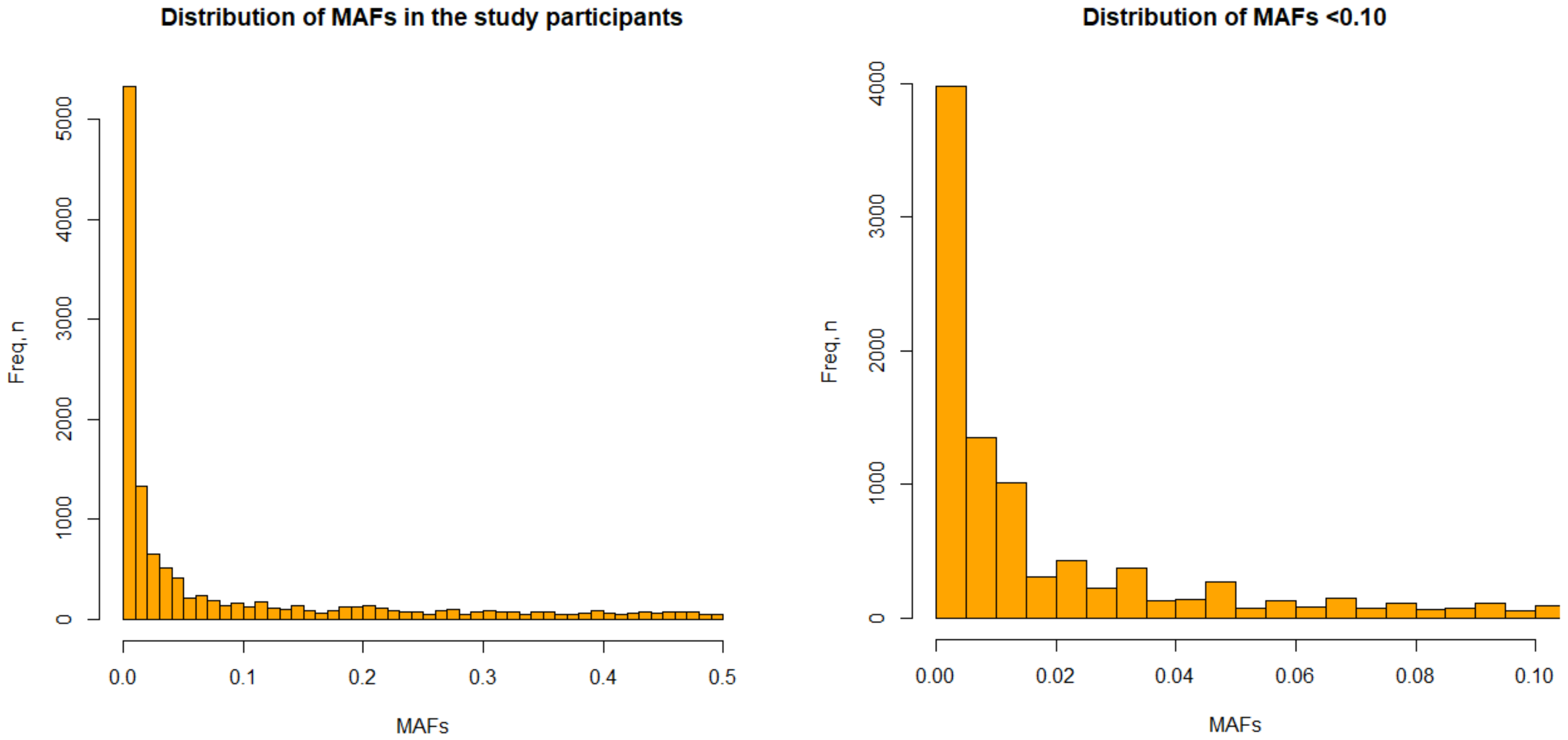


C

**Figure S11. The typical L-shaped distribution of minor allele frequencies (MAFs) of variants in the study participants (Left figure) and limited in MAF <0.10 (Right figure)**

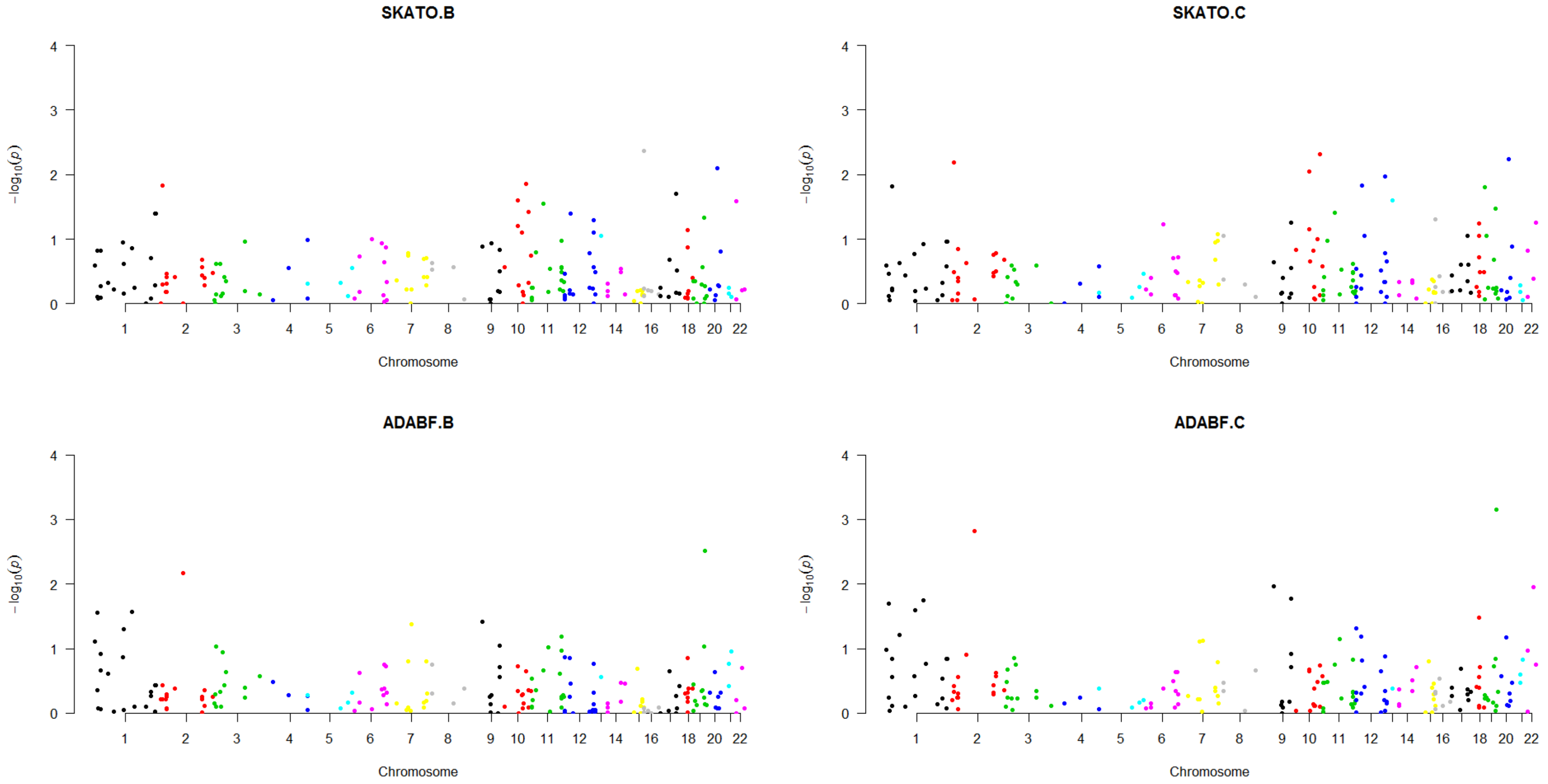


**Figure S12. Manhattan plot of the all SNPs by 4 methods in the all genome-wide level in the study participants**

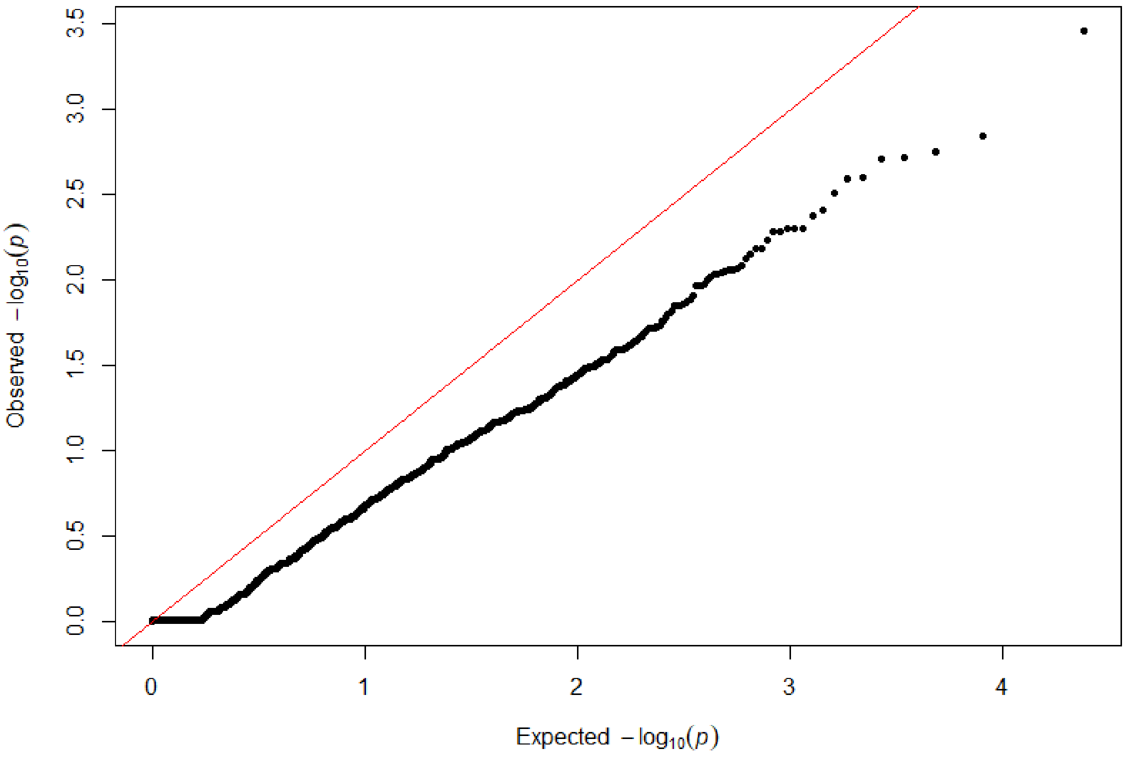


**Figure S13. The Q-Q plot for the FDR or -log10(P) of variants**

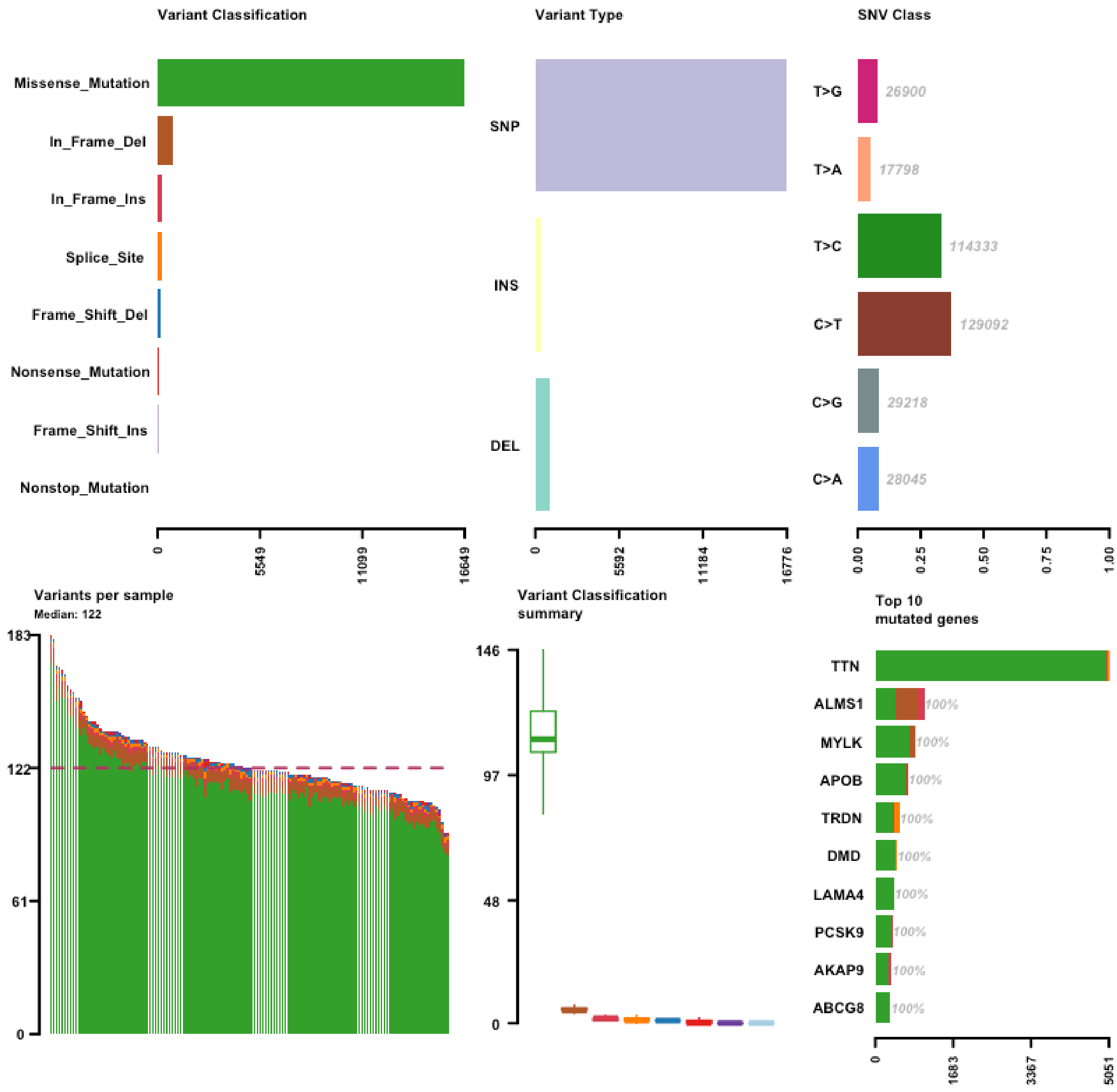


**Figure S14. The summary plot histograms of the variant classification, variant types, SNV class, and the distribution of variants per sample, and the top 10 mutated genes in the study samples**

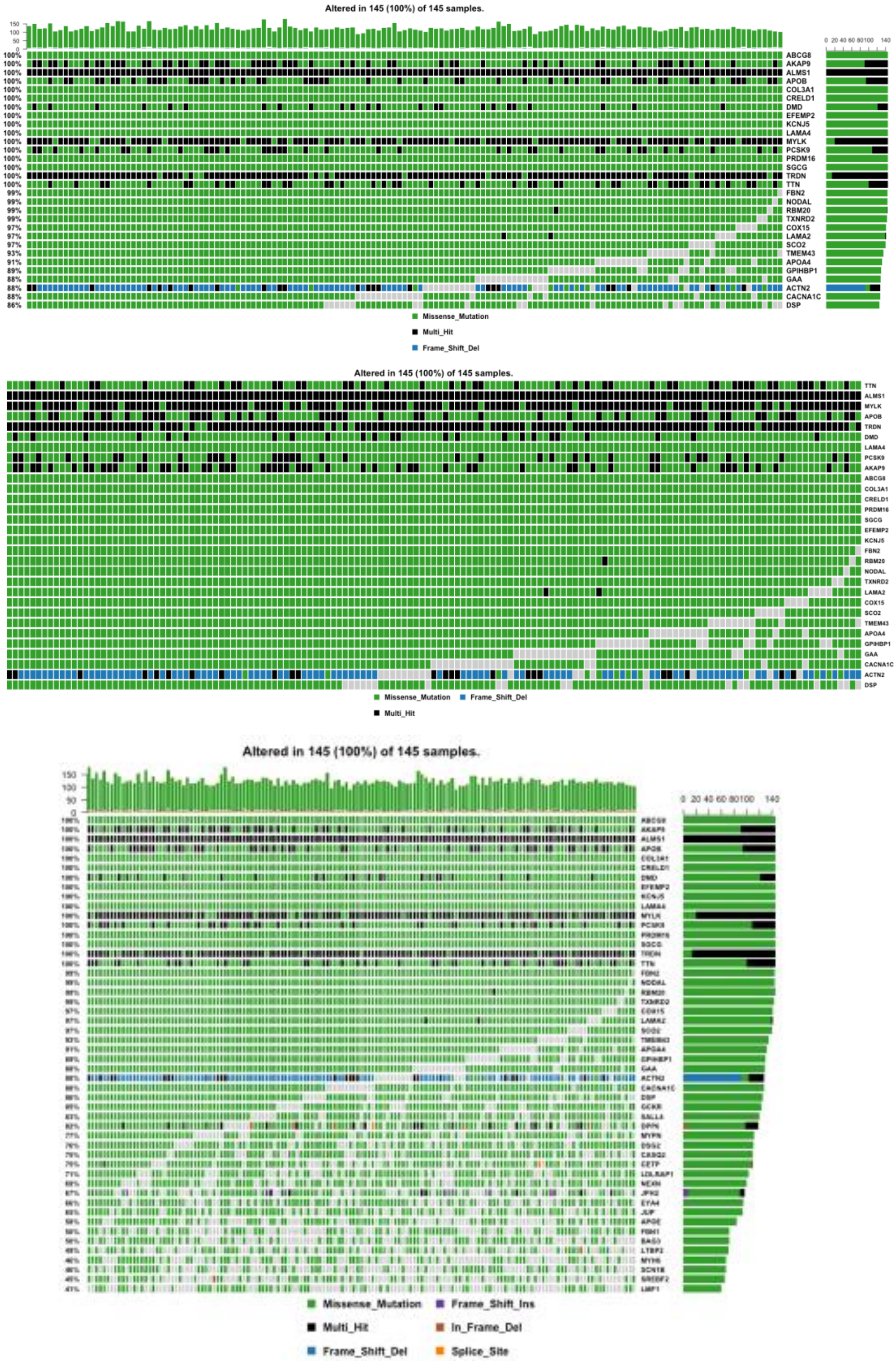


**Figure S15. The onco-plot, onco-strip figures of the various functional mutations of the target genes in the study samples**

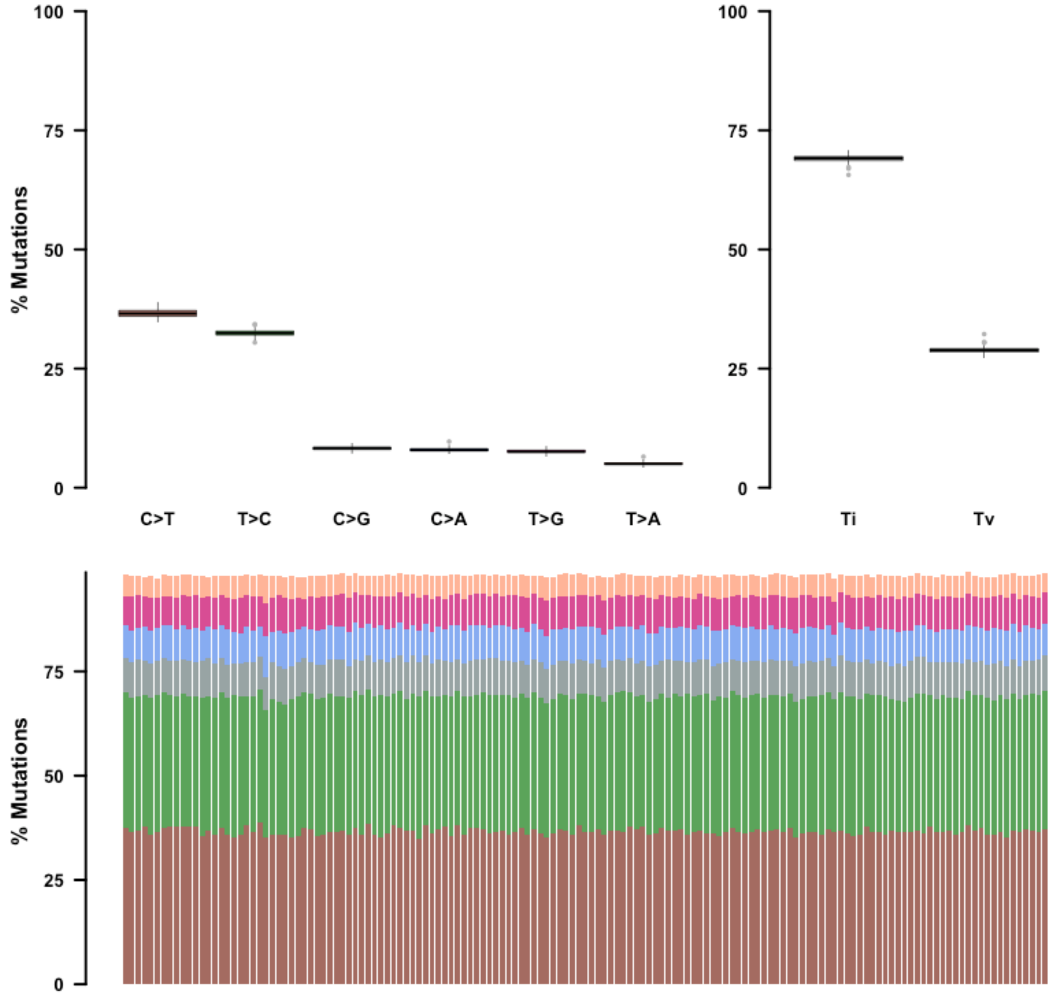


**Figure S16. The plots for titv.png and TTN lolliplot and MYLK lolliplot (TiTv plot)**

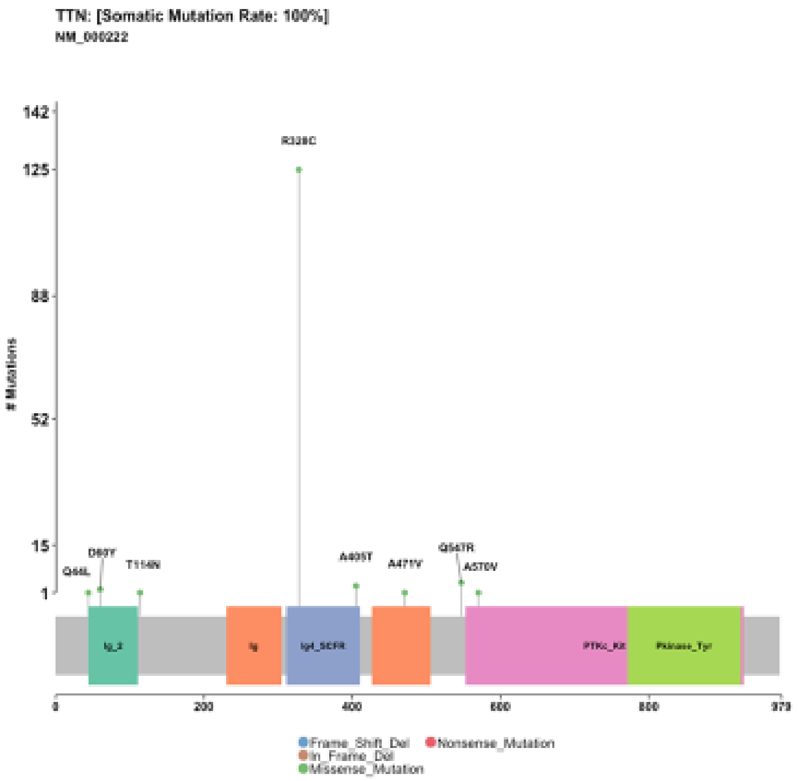


**Figure S17. The plots for titv.png and TTN lolliplot and MYLK lolliplot (the lollipop plot for one target gene, TTN)**

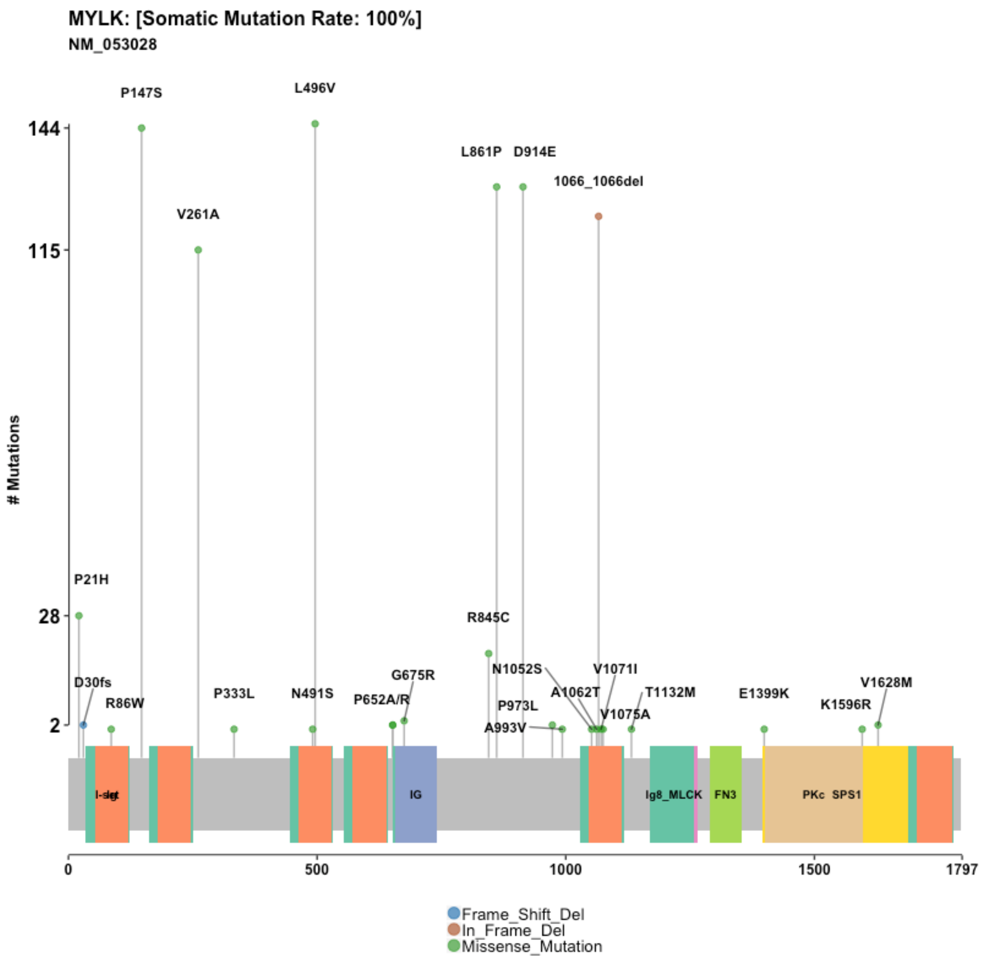


**Figure S18. The plots for titv.png and TTN lolliplot and MYLK lolliplot (lollipop plot for one target gene, MYLK)**

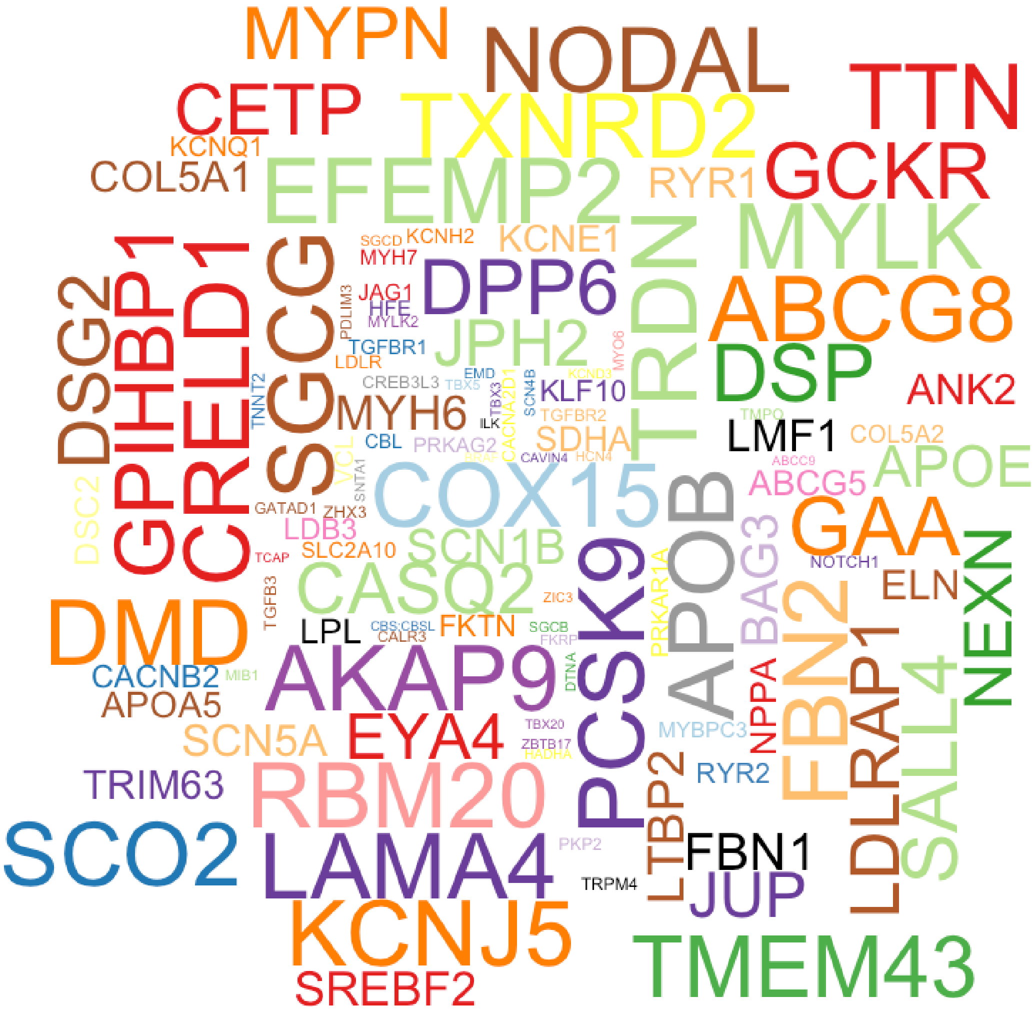


**Figure S19. The genecloud showed the proportions of the variants of target genes in the study samples**

**(A) VCF format**

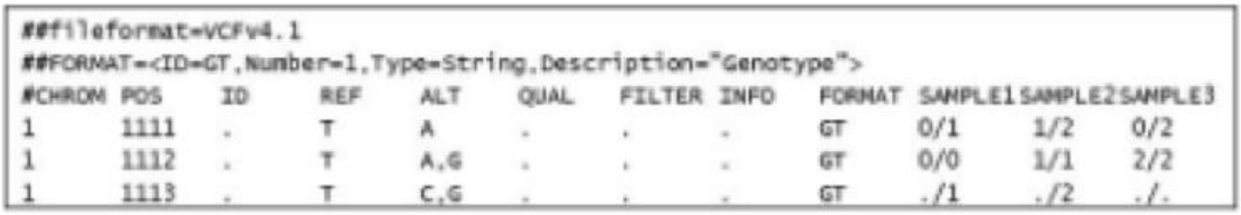


**(B) VCF representation**

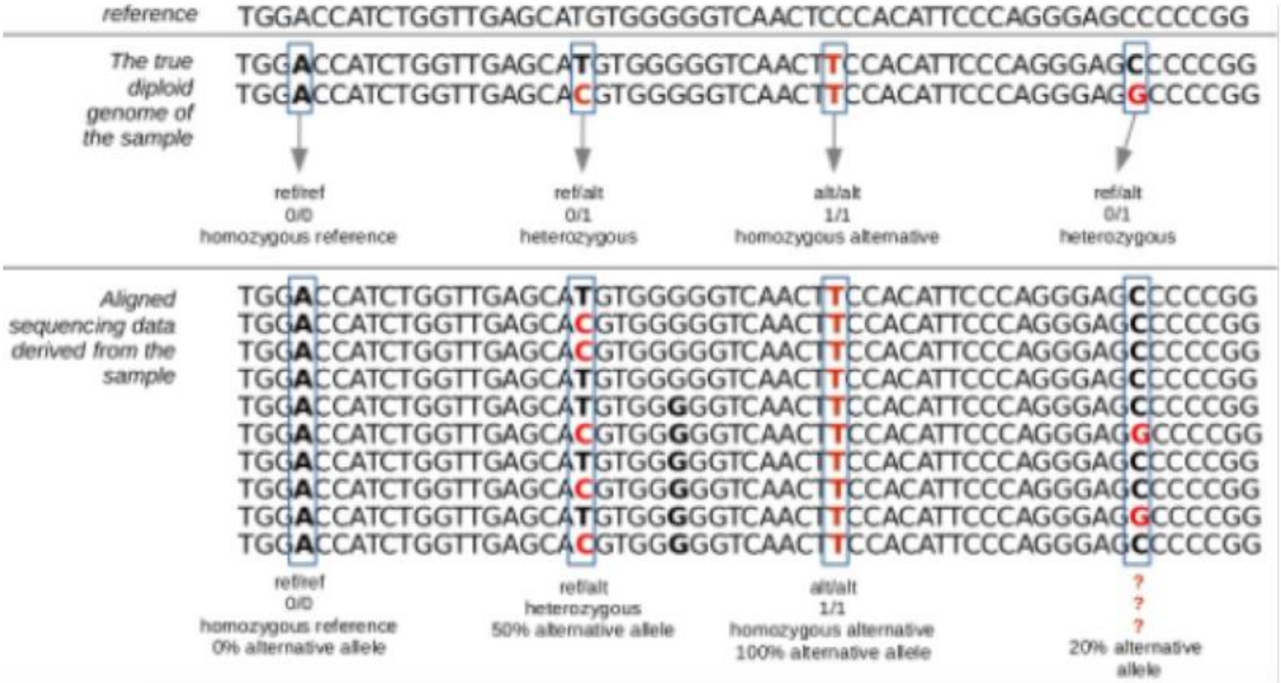


**Figure S20. The information about VCF file**

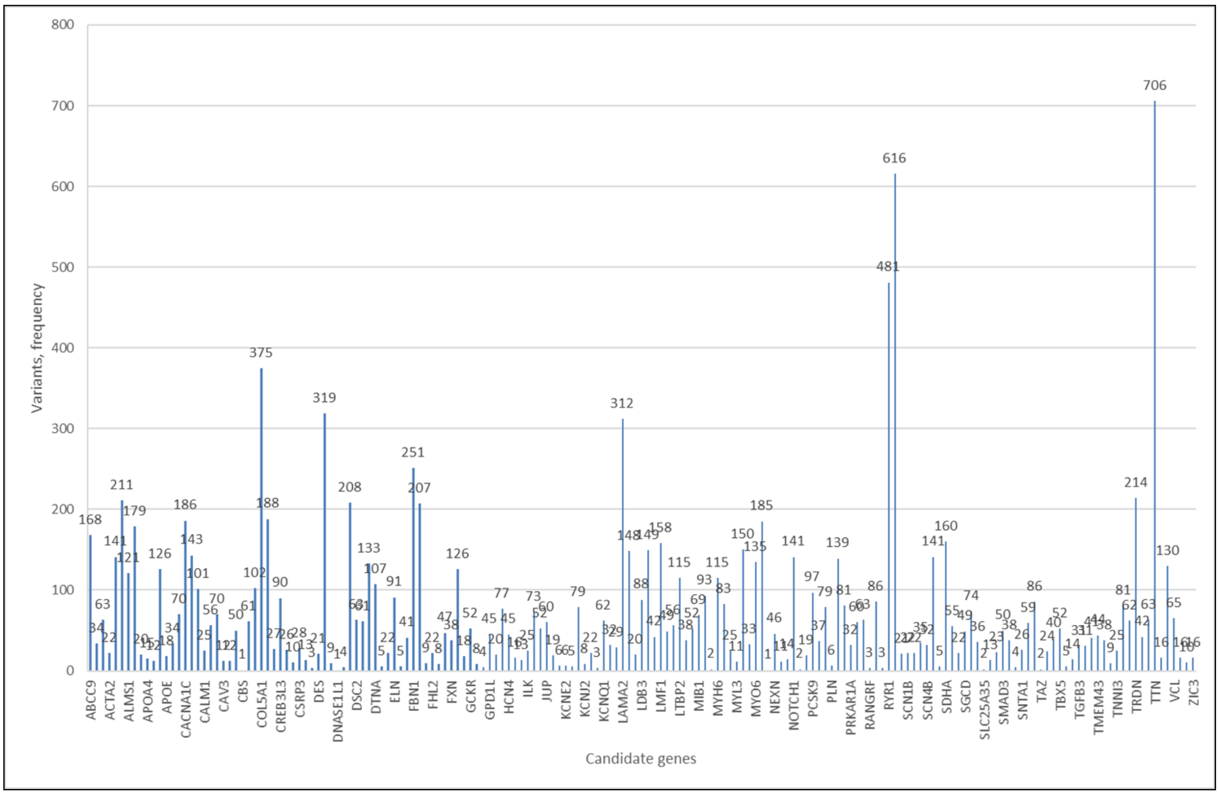


**Figure S21. The distribution of the variant frequency in each target gene in the study population (total 12287 variants and 175 genes)**

## Table S1. The target genes and related frequency and percentage of the study participants.

| Gene | Freq | % | Gene | Freq | % | Gene | Freq | % | Gene | Freq | % | Gene | Freq | % |
|---|---|---|---|---|---|---|---|---|---|---|---|---|---|---|
| ABCC9 | 168 | 1.4 | DCP1B | 3 | 0.0 | JAG1 | 73 | 0.6 | MYO6 | 135 | 1.1 | SHOC2 | 36 | 0.3 |
| ABCG5 | 34 | 0.3 | DES | 21 | 0.2 | JPH2 | 52 | 0.4 | MYPN | 185 | 1.5 | SLC25A35 | 2 | 0.0 |
| ABCG8 | 63 | 0.5 | DMD | 319 | 2.6 | JUP | 60 | 0.5 | NCAPH2 | 1 | 0.0 | SLC25A4 | 13 | 0.1 |
| ACTA2 | 22 | 0.2 | DNAJC19 | 9 | 0.1 | KCND3 | 19 | 0.2 | NEXN | 46 | 0.4 | SLC2A10 | 23 | 0.2 |
| ACTN2 | 141 | 1.2 | DNASE1L1 | 1 | 0.0 | KCNE1 | 6 | 0.1 | NKX2-5 | 11 | 0.1 | SMAD3 | 50 | 0.4 |
| AKAP9 | 211 | 1.7 | DOLK | 4 | 0.0 | KCNE2 | 6 | 0.1 | NODAL | 14 | 0.1 | SMAD4 | 38 | 0.3 |
| ALMS1 | 121 | 1.0 | DPP6 | 208 | 1.7 | KCNE3 | 5 | 0.0 | NOTCH1 | 141 | 1.2 | SNAPC5 | 4 | 0.0 |
| ANK2 | 179 | 1.5 | DSC2 | 63 | 0.5 | KCNH2 | 79 | 0.6 | NPPA | 2 | 0.0 | SNTA1 | 26 | 0.2 |
| ANKRD1 | 20 | 0.2 | DSG2 | 61 | 0.5 | KCNJ2 | 8 | 0.1 | NRAS | 19 | 0.2 | SOS1 | 59 | 0.5 |
| APOA4 | 15 | 0.1 | DSP | 133 | 1.1 | KCNJ5 | 22 | 0.2 | PCSK9 | 97 | 0.8 | SREBF2 | 86 | 0.7 |
| APOA5 | 12 | 0.1 | DTNA | 107 | 0.9 | KCNJ8 | 3 | 0.0 | PDLIM3 | 37 | 0.3 | TAZ | 2 | 0.0 |
| APOB | 126 | 1.0 | DYNC2LI1 | 5 | 0.0 | KCNQ1 | 62 | 0.5 | PKP2 | 79 | 0.6 | TBX20 | 24 | 0.2 |
| APOE | 18 | 0.2 | EFEMP2 | 22 | 0.2 | KLF10 | 32 | 0.3 | PLN | 6 | 0.1 | TBX3 | 40 | 0.3 |
| BAG3 | 34 | 0.3 | ELN | 91 | 0.7 | KRAS | 29 | 0.2 | PRDM16 | 139 | 1.1 | TBX5 | 52 | 0.4 |
| BRAF | 70 | 0.6 | EMD | 5 | 0.0 | LAMA2 | 312 | 2.5 | PRKAG2 | 81 | 0.7 | TCAP | 5 | 0.0 |
| CACNA1C | 186 | 1.5 | EYA4 | 41 | 0.3 | LAMA4 | 148 | 1.2 | PRKAR1A | 32 | 0.3 | TGFB2 | 14 | 0.1 |
| CACNA2D1 | 143 | 1.2 | FBN1 | 251 | 2.0 | LAMP2 | 20 | 0.2 | PTPN11 | 60 | 0.5 | TGFB3 | 31 | 0.3 |
| CACNB2 | 101 | 0.8 | FBN2 | 207 | 1.7 | LDB3 | 88 | 0.7 | RAF1 | 63 | 0.5 | TGFBR1 | 31 | 0.3 |
| CALM1 | 25 | 0.2 | FHL1 | 9 | 0.1 | LDLR | 149 | 1.2 | RANGRF | 3 | 0.0 | TGFBR2 | 41 | 0.3 |
| CALR3 | 56 | 0.5 | FHL2 | 22 | 0.2 | LDLRAP1 | 42 | 0.3 | RBM20 | 86 | 0.7 | TMEM43 | 44 | 0.4 |
| CASQ2 | 70 | 0.6 | FKRP | 8 | 0.1 | LMF1 | 158 | 1.3 | RPL6 | 3 | 0.0 | TMPO | 38 | 0.3 |
| CAV3 | 12 | 0.1 | FKTN | 47 | 0.4 | LMNA | 49 | 0.4 | RYR1 | 481 | 3.9 | TNNC1 | 9 | 0.1 |
| CAVIN4 | 12 | 0.1 | FXN | 38 | 0.3 | LPL | 56 | 0.5 | RYR2 | 616 | 5.0 | TNNI3 | 25 | 0.2 |
| CBL | 50 | 0.4 | GAA | 126 | 1.0 | LTBP2 | 115 | 0.9 | SALL4 | 21 | 0.2 | TNNT2 | 81 | 0.7 |
| CBS | 1 | 0.0 | GATAD1 | 18 | 0.2 | MAP2K1 | 38 | 0.3 | SCN1B | 22 | 0.2 | TPM1 | 62 | 0.5 |
| CETP | 61 | 0.5 | GCKR | 52 | 0.4 | MAP2K2 | 52 | 0.4 | SCN2B | 22 | 0.2 | TRDN | 214 | 1.7 |
| COL3A1 | 102 | 0.8 | GJA5 | 8 | 0.1 | MIB1 | 69 | 0.6 | SCN3B | 35 | 0.3 | TRIM63 | 42 | 0.3 |
| COL5A1 | 375 | 3.1 | GLA | 4 | 0.0 | MYBPC3 | 93 | 0.8 | SCN4B | 32 | 0.3 | TRPM4 | 63 | 0.5 |
| COL5A2 | 188 | 1.5 | GPD1L | 45 | 0.4 | MYH11 | 2 | 0.0 | SCN5A | 141 | 1.2 | TTN | 706 | 5.8 |
| COX15 | 27 | 0.2 | GPIHBP1 | 20 | 0.2 | MYH6 | 115 | 0.9 | SCO2 | 5 | 0.0 | TTR | 16 | 0.1 |
| CREB3L3 | 90 | 0.7 | HADHA | 77 | 0.6 | MYH7 | 83 | 0.7 | SDHA | 160 | 1.3 | TXNRD2 | 130 | 1.1 |
| CRELD1 | 26 | 0.2 | HCN4 | 45 | 0.4 | MYL2 | 25 | 0.2 | SELENON | 55 | 0.5 | VCL | 65 | 0.5 |
| CRYAB | 10 | 0.1 | HFE | 16 | 0.1 | MYL3 | 11 | 0.1 | SGCB | 22 | 0.2 | ZBTB17 | 16 | 0.1 |
| CSRP3 | 28 | 0.2 | HSPB8 | 13 | 0.1 | MYLK | 150 | 1.2 | SGCD | 49 | 0.4 | ZHX3 | 10 | 0.1 |
| CTF1 | 13 | 0.1 | ILK | 25 | 0.2 | MYLK2 | 33 | 0.3 | SGCG | 74 | 0.6 | ZIC3 | 16 | 0.1 |

**Table S2. The summary table for the quality control of the study sample, by the average values of various indicators**

| | Value |
|---|---|
| Average of Total Sequence | 8304739.59 |
| Average of Q30 | 86.44 |
| Average of the percentage of duplicated Reads | 62.31 |
| Average of GC content | 46.86 |

**Table S3. Summarized effect size for the adjusted left ventricular mass, the frequency of selected variants and genes between the case and control groups in the study participants (Treating case/control group as the outcome)**

| Gene | Mode | Variant | rsID number | Estimate | Std. Error | Effect size | P value | control, variant freq (%) | case, variant freq (%) |
|---|---|---|---|---|---|---|---|---|---|
| *COL5A1* | Additive | chr9.134817336_C.G | rs11103537 | -1.1 | 0.3 | -3.6 | 0.0003 | 39.4 | 16.9 |
| | | chr9.134817088_T.G | rs10858282 | -1.2 | 0.3 | -3.5 | 0.001 | 45.6 | 29.2 |
| | | chr9.134818416_G.A | rs3811148 | -1.4 | 0.4 | -3.4 | 0.001 | 25.6 | 10.0 |
| | | chr9.134812212_T.A | rs10745387 | -1.0 | 0.3 | -3.4 | 0.001 | 44.4 | 36.2 |
| | | chr9.134817949_C.T | rs7847840 | -1.1 | 0.3 | -3.3 | 0.001 | 46.9 | 30.8 |
| | | chr9.134810363_G.A | rs3827849 | -1.0 | 0.3 | -3.2 | 0.001 | 45.6 | 39.2 |
| | | chr9.134809545_G.A | rs11103536 | 1.0 | 0.3 | 3.2 | 0.001 | 41.3 | 44.6 |
| | | chr9.134820151_G.C | rs2228560 | -0.9 | 0.3 | -3.1 | 0.002 | 49.4 | 34.6 |
| | | chr9.134814539_A.G | rs4841933 | -0.9 | 0.3 | -3.1 | 0.002 | 45.6 | 39.2 |
| | | chr9.134819115_G.A | rs10745388 | -1.0 | 0.3 | -3 | 0.003 | 49.4 | 37.7 |
| | | chr9.134810354_G.C | rs3827850 | 0.8 | 0.3 | 2.8 | 0.006 | 43.1 | 45.4 |
| | | chr9.134757062_T.G | NF | -2.4 | 0.9 | -2.7 | 0.007 | 7.5 | 1.5 |
| | | chr9.134815988_G.A | rs3827848 | -0.9 | 0.4 | -2.6 | 0.009 | 25.6 | 13.9 |
| | | chr9.134753739_G.A | rs73558066 | -0.7 | 0.3 | -2.5 | 0.013 | 30.6 | 16.9 |
| | | chr9.134816096_T.C | rs4841934 | -0.8 | 0.3 | -2.5 | 0.013 | 46.3 | 44.6 |
| | | chr9.134802186_C.A | rs10858281 | 0.9 | 0.4 | 2.4 | 0.015 | 14.4 | 24.6 |
| | | chr9.134805370_A.G | rs7851471 | 0.9 | 0.4 | 2.4 | 0.017 | 14.4 | 23.9 |
| | | chr9.134809956_A.G | rs3811153 | -0.6 | 0.3 | -2.3 | 0.019 | 48.8 | 39.2 |
| | | chr9.134817934_T.C | rs11792894 | 0.7 | 0.3 | 2.3 | 0.019 | 45.6 | 45.4 |
| | | chr9.134816957_A.G | rs1564479254 | 0.7 | 0.3 | 2.3 | 0.020 | 45.6 | 45.4 |
| | | chr9.134730609_A.C | rs3128600 | -0.7 | 0.3 | -2.3 | 0.021 | 48.8 | 36.9 |
| | | chr9.134817114_A.C | rs10776910 | -0.7 | 0.3 | -2.2 | 0.026 | 49.4 | 42.3 |
| | | chr9.134813552_T.C | rs4841932 | -0.5 | 0.2 | -2.2 | 0.027 | 40.0 | 23.9 |
| | | chr9.134796581_T.G | rs3811162 | 0.8 | 0.3 | 2.2 | 0.029 | 17.5 | 26.2 |
| | | chr9.134699537_A.G | rs7466318 | 1.4 | 0.7 | 2.1 | 0.032 | 2.5 | 10.0 |
| | | chr9.134812893_A.G | rs73664148 | 2.3 | 1.1 | 2.1 | 0.034 | 1.3 | 6.2 |
| | | chr9.134751074_A.G | rs147716718 | -1.4 | 0.7 | -2 | 0.040 | 6.9 | 2.3 |
| | | chr9.134817127_A.G | rs1588584527 | -0.7 | 0.3 | -2 | 0.043 | 49.4 | 43.1 |
| | | chr9.134811167_G.A | rs73664146 | 1.4 | 0.7 | 2 | 0.045 | 3.1 | 7.7 |
| | | chr9.134811840_C.T | rs144759392 | -2.3 | 1.2 | -2 | 0.047 | 5.0 | 0.8 |
| *COL5A1* | Dominant | chr9.134812212_T.A | rs10745387 | -2.0 | 0.5 | -4.1 | 0.00004 | 44.4 | 36.2 |
| | | chr9.134814539_A.G | rs4841933 | -1.9 | 0.5 | -3.8 | 0.0002 | 45.6 | 39.2 |
| | | chr9.134817336_C.G | rs11103537 | -1.5 | 0.4 | -3.7 | 0.0002 | 39.4 | 16.9 |
| | | chr9.134810363_G.A | rs3827849 | -1.7 | 0.5 | -3.6 | 0.0003 | 45.6 | 39.2 |
| *GATAD1* | Dominant | chr7.92448073_C.G | rs6963580 | 1.4 | 0.4 | 3.2 | 0.001 | 16.9 | 27.7 |
| | | chr7.92454268_C.G | rs6465358 | 1.0 | 0.4 | 2.5 | 0.011 | 23.1 | 34.6 |
| | | chr7.92448402_G.A | rs4729043 | 0.9 | 0.4 | 2.2 | 0.031 | 16.3 | 24.6 |
| *LMF1* | Additive | chr16.879428_C.T | rs2076424 | 0.8 | 0.4 | 2.2 | 0.027 | 21.9 | 27.7 |
| *LMF1* | Dominant | chr16.868783_G.C | rs1035269256 | 2.3 | 1.2 | 2 | 0.049 | 0.6 | 3.8 |
| *PTPN1* | Dominant | chr12.112457337_G.A | rs11066315 | 0.9 | 0.4 | 2.2 | 0.026 | 39.4 | 45.4 |
| | | chr12.112504549_G.A | rs4767860 | 0.9 | 0.4 | 2.1 | 0.040 | 39.4 | 44.6 |
| *SCN4B* | Recessive | chr11.118141726_C.A | rs11604096 | 1.1 | 0.4 | 2.8 | 0.006 | 34.4 | 45.4 |
| *SGCG* | Recessive | chr13.23295768_T.C | rs4411362 | 1.3 | 0.5 | 2.3 | 0.019 | 30.6 | 40.0 |
| *SREBF2* | Recessive | chr22.41873624_C.T | rs133291 | 1.9 | 0.6 | 3.4 | 0.001 | 43.1 | 42.3 |

**Table S4. Summarized effect size for the adjusted left ventricular mass, the frequency of selected variants and genes between the case and control groups in the study participants (Treating outcome as the adjusted left ventricular mass)**

| Gene | Mode | Variant | Estimate | Std. Error | Effect size | P value | control, variant freq (%) | case, variant freq (%) |
|---|---|---|---|---|---|---|---|---|
| *RBM20* | Additive | chr10.110727905_G.A | 230.3 | 67.7 | 3.4 | 0.001 | 0.0 | 1.5 |
| | | chr10.110727911_C.T | 230.3 | 67.7 | 3.4 | 0.001 | 0.0 | 1.5 |
| | | chr10.110831376_A.G | 62.7 | 25.4 | 2.5 | 0.015 | 3.8 | 7.7 |
| | | chr10.110783929_C.G | 150.7 | 62.5 | 2.4 | 0.017 | 0.6 | 3.1 |
| | | chr10.110823999_C.T | -27.3 | 13.1 | -2.1 | 0.039 | 45.0 | 30.0 |
| | | chr10.110821836_G.A | 285.5 | 140.4 | 2 | 0.044 | 0.0 | 0.8 |
| | | chr10.110811936_G.C | -41.8 | 20.7 | -2 | 0.045 | 17.5 | 10.0 |
| *COL5A1* | Additive | chr9.134817336_C.G | -59.4 | 15.1 | -3.9 | 0.0001 | 39.4 | 16.9 |
| | | chr9.134812212_T.A | -55.8 | 15.6 | -3.6 | 0.0005 | 44.4 | 36.2 |
| | | chr9.134818416_G.A | -69.2 | 20.0 | -3.5 | 0.001 | 25.6 | 10.0 |
| | | chr9.134817088_T.G | -59.1 | 17.6 | -3.4 | 0.001 | 45.6 | 29.2 |
| | | chr9.134809545_G.A | 56.0 | 16.8 | 3.3 | 0.001 | 41.3 | 44.6 |
| | | chr9.134814539_A.G | -53.1 | 16.6 | -3.2 | 0.002 | 45.6 | 39.2 |
| | | chr9.134817949_C.T | -53.3 | 17.1 | -3.1 | 0.002 | 46.9 | 30.8 |
| | | chr9.134810363_G.A | -49.9 | 16.6 | -3 | 0.003 | 45.6 | 39.2 |
| | | chr9.134819115_G.A | -50.0 | 17.2 | -2.9 | 0.004 | 49.4 | 37.7 |
| | | chr9.134757062_T.G | -111.2 | 39.1 | -2.8 | 0.005 | 7.5 | 1.5 |
| | | chr9.134820151_G.C | -46.6 | 16.6 | -2.8 | 0.006 | 49.4 | 34.6 |
| | | chr9.134810354_G.C | 45.4 | 17.0 | 2.7 | 0.009 | 43.1 | 45.4 |
| | | chr9.134699537_A.G | 80.3 | 31.6 | 2.5 | 0.012 | 2.5 | 10.0 |
| | | chr9.134753739_G.A | -43.0 | 17.2 | -2.5 | 0.014 | 30.6 | 16.9 |
| | | chr9.134816096_T.C | -46.0 | 18.5 | -2.5 | 0.014 | 46.3 | 44.6 |
| | | chr9.134817934_T.C | 43.6 | 18.0 | 2.4 | 0.017 | 45.6 | 45.4 |
| | | chr9.134816957_A.G | 43.4 | 18.2 | 2.4 | 0.018 | 45.6 | 45.4 |
| | | chr9.134750722_C.A | 230.8 | 97.5 | 2.4 | 0.019 | 0.0 | 1.5 |
| | | chr9.134761733_T.C | 230.8 | 97.5 | 2.4 | 0.019 | 0.0 | 1.5 |
| | | chr9.134642512_C.G | 191.9 | 82.8 | 2.3 | 0.022 | 0.0 | 2.3 |
| | | chr9.134817114_A.C | -43.2 | 18.7 | -2.3 | 0.022 | 49.4 | 42.3 |
| | | chr9.134754012_G.A | 132.5 | 57.8 | 2.3 | 0.023 | 0.6 | 3.9 |
| | | chr9.134815988_G.A | -46.1 | 20.4 | -2.3 | 0.026 | 25.6 | 13.9 |
| | | chr9.134813552_T.C | -31.1 | 14.2 | -2.2 | 0.031 | 40.0 | 23.9 |
| | | chr9.134817127_A.G | -41.1 | 18.9 | -2.2 | 0.031 | 49.4 | 43.1 |
| | | chr9.134753692_C.G | -28.2 | 13.2 | -2.1 | 0.035 | 43.1 | 30.8 |
| | | chr9.134753693_C.T | -28.2 | 13.2 | -2.1 | 0.035 | 43.1 | 30.8 |
| | | chr9.134730532_T.A | 317.7 | 149.3 | 2.1 | 0.035 | 0.0 | 0.8 |
| | | chr9.134803390_T.C | 317.7 | 149.3 | 2.1 | 0.035 | 0.0 | 0.8 |
| | | chr9.134803393_T.G | 317.7 | 149.3 | 2.1 | 0.035 | 0.0 | 0.8 |
| | | chr9.134809956_A.G | -32.2 | 15.1 | -2.1 | 0.035 | 48.8 | 39.2 |
| | | chr9.134812893_A.G | 79.9 | 37.6 | 2.1 | 0.035 | 1.3 | 6.2 |
| | | chr9.134797080_C.T | -48.0 | 23.0 | -2.1 | 0.039 | 18.1 | 13.1 |
| | | chr9.134802186_C.A | 40.5 | 19.8 | 2 | 0.043 | 14.4 | 24.6 |
| *COL5A1* | dominant | chr9.134812212_T.A | -110.3 | 24.4 | -4.5 | 0.00001 | 44.4 | 36.2 |
| | | chr9.134814539_A.G | -103.1 | 25.6 | -4 | 0.0001 | 45.6 | 39.2 |
| | | chr9.134817336_C.G | -85.6 | 21.7 | -3.9 | 0.0001 | 39.4 | 16.9 |
| | | chr9.134809956_A.G | -85.3 | 23.6 | -3.6 | 0.0004 | 48.8 | 39.2 |
| *GATAD1* | dominant | chr7.92448073_C.G | 69.9 | 23.9 | 2.9 | 0.004 | 16.9 | 27.7 |
| | | chr7.92454268_C.G | 55.0 | 23.5 | 2.3 | 0.021 | 23.1 | 34.6 |
| | | chr16.879711_C.T | 0.8 | 0.3 | 2.3 | 0.019 | 20.6 | 26.9 |
| *LMF1* | Additive | chr16.870043_A.G | 325.8 | 97.9 | 3.3 | 0.001 | 0.0 | 1.5 |
| | | chr16.855520_C.A | -155.1 | 52.9 | -2.9 | 0.004 | 4.4 | 0.0 |
| | | chr16.855540_C.T | -155.1 | 52.9 | -2.9 | 0.004 | 4.4 | 0.0 |
| | | chr16.855563_A.G | -155.1 | 52.9 | -2.9 | 0.004 | 4.4 | 0.0 |
| | | chr16.855016_G.T | -159.7 | 57.4 | -2.8 | 0.006 | 3.8 | 0.0 |
| | | chr16.855031_A.G | -159.7 | 57.4 | -2.8 | 0.006 | 3.8 | 0.0 |
| | | chr16.855604_C.A | -159.7 | 57.4 | -2.8 | 0.006 | 3.8 | 0.0 |
| | | chr16.869667_C.T | -159.7 | 57.4 | -2.8 | 0.006 | 3.8 | 0.0 |
| | | chr16.870071_C.T | -159.7 | 57.4 | -2.8 | 0.006 | 3.8 | 0.0 |
| | | chr16.870310_C.G | -159.7 | 57.4 | -2.8 | 0.006 | 3.8 | 0.0 |
| | | chr16.870571_G.A | -159.7 | 57.4 | -2.8 | 0.006 | 3.8 | 0.0 |
| | | chr16.871144_C.A | -159.7 | 57.4 | -2.8 | 0.006 | 3.8 | 0.0 |

| | | | | | | | | |
|---|---|---|---|---|---|---|---|---|
| | | chr16.880161_T.C | 363.8 | 139.5 | 2.6 | 0.010 | 0.0 | 0.8 |
| | | chr16.869442_T.C | -125.8 | 50.0 | -2.5 | 0.013 | 3.8 | 0.0 |
| | | chr16.879606_C.T | -104.7 | 49.1 | -2.1 | 0.035 | 4.4 | 0.8 |
| *LMF1* | dominant | chr16.870043_A.G | 325.8 | 97.9 | 3.3 | 0.001 | 0.0 | 1.5 |
| | | chr16.855520_C.A | -155.1 | 52.9 | -2.9 | 0.004 | 4.4 | 0.0 |
| *PTPN1* | dominant | chr12.112457457_A.G | 326.0 | 95.2 | 3.4 | 0.001 | 0.0 | 1.5 |
| | | chr12.112457544_T.G | 326.0 | 95.2 | 3.4 | 0.001 | 0.0 | 1.5 |
| *SCN4B* | Recessive | chr11.118141726_C.A | 51.1 | 24.0 | 2.1 | 0.035 | 34.4 | 45.4 |
| *SGCG* | Recessive | chr13.23279852_C.A | 460.6 | 135.4 | 3.4 | 0.001 | 0.0 | 1.5 |
| *SREBF2* | Recessive | chr22.41873624_C.T | 118.9 | 28.0 | 4.2 | 0.00004 | 43.1 | 42.3 |

**Table S5. The distribution of the functional change of variants in the study participants**

| Function | Frequency | % |
|---|---|---|
| UTR3 | 394 | 3.2 |
| UTR5 | 208 | 1.7 |
| Exonic | 1804 | 14.7 |
| Intronic | 9875 | 80.4 |
| Splicing | 6 | 0.1 |
